# Pyroptosis-Related Gene Signatures Enable Robustly Diagnosis, Prognosis and Immune Responses Prediction in Uterine Corpus Endometrial Carcinoma

**DOI:** 10.7150/jca.104826

**Published:** 2025-05-08

**Authors:** Xuanming Chen, Xiangyu Jin, Jiafu Wang, Hanfei Li, Chuanfang Wu, Jinku Bao

**Affiliations:** Key Laboratory of Bio-Resource and Eco-Environment of Ministry of Education, Collage of Life Science, Sichuan University, Chengdu, China

**Keywords:** pyroptosis, uterine corpus endometrial carcinoma, diagnosis, prognostic model, immunotherapy.

## Abstract

**Purpose:** Uterine corpus endometrial carcinoma (UCEC) is a gynecological malignancy with poor prognosis and high lethality rates. Pyroptosis, a pro-inflammatory programmed cell death pattern, significantly influences tumor growth, development, and metastasis. We intend to explore whether pyroptosis-related genes can be screened as targets for early detection and patient prognosis.

**Methods:** We used nine common machine learning algorithms to build classifiers based on the pyroptosis-related genes, evaluated the classifiers' performance using metrics like the receiver operating characteristic curve (ROC), and verified the results using external datasets. Using Least Absolute Shrinkage and Selection Operator (LASSO) regression analysis, we built a predictive model. ROC and univariate/multivariate Cox analyses were used to assess the model's performance and its independence in predicting patient prognosis. We used a variety of statistical methods and algorithms to investigate the connection between tumor immunity and pyroptosis-related genes.

**Results:** We identified 26 pyroptosis-related genes associated with the diagnosis and prognosis of UCEC. We found the logistic regression classifier performing the best. We then constructed a predictive model based on seven PRGs about *IRF2, TIRAP, BAK1, GSDMD, CHMP2A, GPX4, CHMP2B*. The pyroptosis-related gene risk signature (PRGRS) effectively classified UCEC patients. We demonstrated that PRGRS independently impacted UCEC prognosis and confirmed its expression using qRT-PCR experiments. Furthermore, we found associations between PRGRS and tumor immune response.

**Conclusion:** Our study highlights novel pyroptosis-related gene signatures that may be utilized for early screening and prognosis prediction in UCEC patients, offering potential targets for future research and guidance for personalized anticancer therapies.

## 1. Introduction

One of the top 10 most prevalent cancers worldwide, uterine corpus endometrial carcinoma (UCEC) is women's second most frequent gynecological tumor 1. In the United States alone, the incidence and mortality rates for UCEC in 2024 were estimated to be 67,880 and 13,250, respectively. Moreover, unlike for many other cancers, the endometrial cancer (EC) survival rates have not increased over time 1,2. Early-stage EC patients often have a good prognosis following surgery, while ones with progressed, metastatic, or recurrent EC frequently do not 3-5. Indirectly, the high rate of EC has been caused by the lack of prognostic and early diagnostic methods. Traditional clinical staging provides inaccurate clinical prognostic information, and some patients within the same subgroup may present with varying clinical outcomes 6. Therefore, to increase the precision of early diagnosis and prognosis prediction, it is necessary to employ legitimate, reliable, and sensitive markers to identify the various phases of UCEC.

Pyroptosis is a type of pro-inflammatory necrosis, i.e., programmed cell death that is not typical 7. Gasdermin (GSDM) family proteins (designated GSDM A-F) play an essential part in the pyroptosis process by mediating the formation of cellular pores, which induces cell swelling, plasma membrane burst, and inflammatory content unleashing, promoting a range of inflammatory and immunological responses 8,9. Numerous pathological stimuli initiate the canonical inflammasome pathway of pyroptosis, which results in the kinase of caspase-1 and the release of inflammatory vesicles. This event, in turn, promotes GSDMD cleavage and matures pro-inflammatory cytokines such as interleukin (IL)-1β and IL-18. Then the GSDMD N-terminal structural domain creates a pore in the plasma membrane, which eventually undergoes osmotic cleavage to release intracellular inflammatory substances 10. Two other pathways are also involved in pyroptosis: 1) the caspase-4/5/11-dependent noncanonical inflammasome pathway; and 2) a caspase-3-regulated pathway, in which caspase-3 cleaves GSDME to promote pore formation and trigger pyroptosis 11,12. Pyroptosis was first identified as a crucial defense mechanism against infection; subsequent research revealed that pyroptosis is also strongly linked to atherosclerosis, inflammatory bowel disease, and diabetic nephropathy 13-15. Moreover, it has been demonstrated that pyroptosis facilitates the growth of neoplasms 16.

The mechanisms of pyroptosis in tumor cells have been the subject of several studies, but the function of the role of pyroptosis in the progression of tumors was ambiguous 17. Most studies point to the dual involvement of pyroptosis for cancer development 18-20. Pyroptosis results in the inflammatory cytokines unleashing, which, together with the tumor microenvironment (TME), impact tumor growth and invasion. Meanwhile, the induction of cellular pyroptosis results in tumor cell death and limits tumor development. Pyroptosis has been linked to tumor cell invasion and metastasis, and it has also been shown to affect how well chemotherapeutic drugs work 21-24. The presence of a large number of infiltrating immune cells and inflammatory cytokines in endometrial cancer tissues suggests a strong link between endometrial cancer and pyroptosis 25. Contemporary research discovered that proteins linked with pyroptosis were expressed in EC. Hydrogen induces reactive oxygen species (ROS) production and regulates the Mitosox/NLRP3/Caspase-1/GSDMD signaling pathway, which results in EC cell pyrotosis. This inhibits tumor development ultimately 26. Several studies have reported that pyroptosis-related genes can serve as prognostic signatures for various cancers, including breast 27, bladder 28, gastric 29,30, lung 31, liver 32, and skin 33 cancers. However, although the potential mechanism of pyroptosis in UCEC has been delineated, the capacity of pyroptosis in the diagnosis and prognosis of UCEC is still unclear. The diagnostic quality of pyroptosis-related genes (PRGs) in identifying UCEC and normal tissues was uncovered using machine learning (ML) algorithms.

Immunotherapy works by decreasing negative immunological regulation and stimulating the immune system to remove tumor cells 34,35. pembrolizumab and dostarlimab have been clinically validated and FDA-approved for the treatment of patients with endometrial cancer, and durvalumab and combination therapy are currently in clinical trials 36-39. The difficulty for immunotherapy is to identify highly immunogenic patients' potential to respond, as well as to elicit or improve immunogenicity in immune exhaustion tumors 40. The discovery of novel biomarkers can aid in the selection of the best treatment for patients.

We constructed a new prognostic risk signature of PRGs in UCEC. We then analyzed the performance of this risk model in regarding tumor immune infiltration, chemotherapy, and immunotherapy in UCEC patients. Our research suggests a fresh perspective on pyroptosis' function in UCEC and provides PRG-based risk signatures, which can be applied to the early diagnosis, prognosis, and immunological reaction anticipation of UCEC patients as well as the tailoring of anti-cancer therapy.

## 2. Materials and methods

### 2.1 Data gathering and integration

The RNA sequencing information and clinical data for 560 patients with UCEC were obtained from the TCGA database (https://portal.gdc.cancer.gov/) 41. The exclusion criteria were as follows: a. Primary site was corpis uteri; b. Disease types were (adenomas and adenocarcinomas) and (cystic, mucinous and serous neoplasms); c. Primary solid tumors (sample ID ending with -01A) and Solid Tissue Normal (sample ID ending with -11A) samples are required. A total of 532 tumor samples and 23 normal samples were filtered. Transcriptome profiles for validation were collected in GEO (https://www.ncbi.nlm.nih.gov/geo/) such as GSE63678 and GSE17025 42,43. RNA-seq data for 78 normal uterine samples were obtained in GTEx (https://xenabrowser.net/datapages/) 44. RNA-seq information from TCGA-UCEC and GTEx-Uterine datasets had been integrated and standardized by implementing the R package “limma”.

### 2.2 Differentially expressed PRGs for identification

61 Pyroptosis-related Genes (PRGs), presented in Table S1, had been recognized from the GeneCards database (https://www.genecards.org) through exploration of the key term "pyroptosis", the MsigDB database (https://www.gsea-msigdb.org/gsea/msigdb/index.jsp), as well as previously published papers 33,45-47. Differentially expressed genes (DEGs) between UCEC samples and adjacent non-tumor samples were identified by the false discovery rate (FDR)-adjusted p-value (i.e., q-value) ≤ 0.05 and |log2 fold change (FC)| > 1 via the “limma”R package. The "corrplot" R package has been adopted to assess correlations involving differentially expressed PRGs (DEPRGs). The protein-protein interaction (PPI) network for differentially expressed genes (DEGs) was constructed employing the STRING v11.5 (https://string-db.org).

### 2.3 DEPRGs-based classifiers for UCEC diagnosis: design and assessment

We first integrated the TCGA-UCEC and GTEx-Uterine datasets, extracted the expression amounts of differentially expressed genes related to focal death in each sample, and then counted and cleaned the data for missing values and null values, resulting in a total of 281 samples in the integrated results, which included 101 normal tissue samples and 180 tumor tissue samples. Subsequently, the dataset was randomly divided into the training set (including 67 normal tissue samples and 130 tumor tissue samples with a total of 197 transcriptome samples) and the test set (including 34 normal tissue samples and 50 tumor tissue samples with a total of 84 transcriptome samples) in the ratio of 7:3 according to the stratified sampling method. The training set data were trained with the following models for the classifier: k-nearest neighbor (k-NN), logistic regression (LR), support vector machine (SVM), artificial neural network (ANN), decision tree (DT), random forest (RF), XGBoost, LightGBM and CatBoost. These models are implemented in the following R package: "caret", "e1071", "nnet", "rpart", "lightgbm", "xgboost", and "catBoost" 48-50. We employed a 10-fold cross-validation of the training set stratified by group to discover the hyperparameter space of each model to ensure that the model has strong generalization ability.

The pyroptosis-regulated gene (PRG)-based classifiers were assessed with the support of the R package "caret" and "ROCR". We evaluated those models' predictive performance via the following parameters: accuracy, precision, recall, F1-score, and area under the receiver operating characteristic curve (AUC). The testing set was used for internal review. Data from GSE63678 (validating set 1) and GSE17025 (validating set 2) were utilized for external validation. By using "scale" function, the data for each group were normalized. Supplemental Table S2 lists the key parameters utilized in the algorithms mentioned above.

### 2.4 Consensus clustering

The concordance matrix and cumulative distribution function (CDF) were utilized to establish the perfect quantity of isoforms in concurrence clustering. Consequently, in line with the transcriptional matrix of DEPRGs, two individual clusters (clusters A and B) were produced utilizing the "ConsensusClusterPlus" R package 51. The "prcomp" function found in the "stats" R package was applied for the performance of cluster-based principal component analysis (PCA). The "tsne" R package is utilized to depict the clustering outcomes and to evaluate them by utilizing t-distributed stochastic neighbor embedding (t-SNE). Predicated on the chi-square tests and the "survival" R package, we explored the link between clusters and patients' clinical features. Adopting R packages "pheatmap," "survival," and "survminer," respectively, findings were displayed as heatmaps and Kaplan-Meier (K-M) curves.

### 2.5 PRGRS: construction and evaluation

To further pinpoint DEPRGs connected to prognosis, univariate Cox regression analysis was used. With the aim of minimizing the number of candidate genes and building a more accurate prognostic model, LASSO Cox regression investigations had been carried out with R package "glmnet" 52. The "survival" R package was adopted for univariate Cox regression analysis with an FDR limit of 0.05. The dependent factors in the regression were patients' overall survival and state within the TCGA cohort, and the independent variables were the normalized expression matrix of the putative prognostic DEPRG. Data from the TCGA-UCEC dataset were randomly allocated to 70% of the subjects as the training set by using a stochastically generated sequence; the entire group was then utilized as a validation dataset. The penalty parameter (λ) for gene signatures was computed using tenfold cross-validation with the minimum criteria. (Namely, the value corresponding to the slightest partial likelihood deviation). The correlation coefficients of the seven top genes were computed after they had been filtered. Then, its relevant risk score was computed utilizing below the formula:



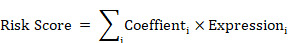



Entire UCEC individuals were sorted into high- and low-risk subgroups through the median risk score. The "survival" and "survminer" R packages were applied to perform a log-rank test on the differing overall survival rates among high- and low-risk sub-categories. Utilizing the R package "survivalROC" a time-dependent receiver operating characteristic (ROC) study was conducted to ascertain the model's prognosis sensitivity and specificity. Gene expression, the landscape of risk scores, and the survival state were also considered. For risk scores and clinical factors in the first, third, and fifth years, the area under the receiver operating characteristic curve (AUC) was computed, correspondingly. Univariate and multivariate Cox regression analysis evaluated the link and independence between the risk scores and patients' prognosis. Nomogram illustrates the survival rate at 1, 3, and 5 years for the entire UCEC dataset was then calculated by integrating the five clinical features of age, cancer stage, tumor grade, histological type, and risk score. Calibration curves were adopted to assess the established nomogram reliability 53.

### 2.6 qRT-PCR validation

The following stated the cells and the culture conditions: Two UCEC cellular lines (Ishikawa and HEC-1-B) 54-56 and one human endometriosis cellular strain hEM15A (National Biomedical Experimental Cell Repository, China) had been prepared DMEM with 10% FBS at 37°C in a 5% CO2 incubator. All cellular lines were obtained in March 2023. The total RNA from these cells was obtained using AG RNAex Pro Reagent (Accurate Biology, China), and cDNA was synthesized with a Hifair® III 1st Strand cDNA Synthesis Kit (gDNA Digester Plus, YEASEN, China). A Hieff UNICON® Universal Blue qPCR SYBR Green Master Mix (YEASEN, China) was applied for the qPCR. A catalogue of primer sequences is provided in Supplementary Table S3.

### 2.7 Function enrichment analyses

DAVID (https://david.ncifcrf.gov) 57 was utilized to execute Gene Ontology (GO) classification, and the Kyoto Encyclopedia of Genes and Genomes (KEGG) was employed to scrutinize the biological processes or pathways that differentially expressed genes (DEGs) partake in. DEGs were filtered into subgroups of high risk compared to those of low risk. Gene set enrichment analysis (GSEA) was implemented to investigate the roles and pathways for various subgroups 58,59. The "ggplot2" R package was used in illustrating outcomes. Using the R package "gsva," the single-sample gene set enrichment analysis algorithm (ssGSEA) was estimated. After that, 13 immune-related pathway activation and 16 immune cell-type infiltration were measured.

### 2.8 Drug sensitivity prediction

GDSC (https://www.cancerxgene.org/) 60 database was used to estimate each sample's susceptibility to pharmacotherapy. The half-maximal inhibitory concentration (IC50) for the instances was quantified using the R package "pRRophetic," where ridge regression was applied. Pearson correlation analysis explored the correlation between the signature expression and the samples' compound sensitivity.

### 2.9 Mutation analysis

TCGA provided the mutation materials for UCEC. The format of somatic mutation data is mutation annotation (MAF). Each patient's tumor mutational burden (TMB) value was determined in this study, and the correlation between TMB and risk signature was studied 61.

### 2.10 Immune infiltration analysis

TIMER 2.0 (http://timer.cistrome.org/) 62,63. The associations between risk scores and the immunological cell qualities of UCEC individuals were assessed using the algorithms TIMER, CIBERSORT, CIBERSORT-ABS, QUANTISEQ, MCPcounter, XCELL, and EPIC (using data from TCGA). The relevant immune, stroma, and ESTIMATE scores were assessed utilizing the ESTIMATE algorithm 64. The CIBERSORT platform then quantified the proportions of the 22 immune cell types in UCEC patients.

### 2.10 ICI analysis

Immune checkpoint inhibitors (ICIs) inhibit the checkpoint activity of T cells and have been shown to be effective as a cancer therapy, especially CTLA-4 and PD-1 65. The four primary factors in assessing tumor immunogenicity are immunomodulatory agents, major histocompatibility complex (MHC) molecules, executing cells, and immune-inhibitory cells. By evaluating the expression degrees of the genes related to these four factors, we can derive a patient's immunophenoscore (IPS). IPS is scored from 0 to 10, whereby score size is proportional to immunogenicity. IPSs (including IPS-CTLA-4, IPS-CTLA-4/PD-1/PD-L1/PD-L2, and IPS-PD-1/PD-L1/PD-L2) were obtained at TCIA (https://tcia.at/home) 66. IPS helps reflect the effectiveness of the ICI treatment for UCEC patients. Then, it was examined at how risk scores and ICI expression related.

### 2.11 Statistical analysis

**Figure 1:**
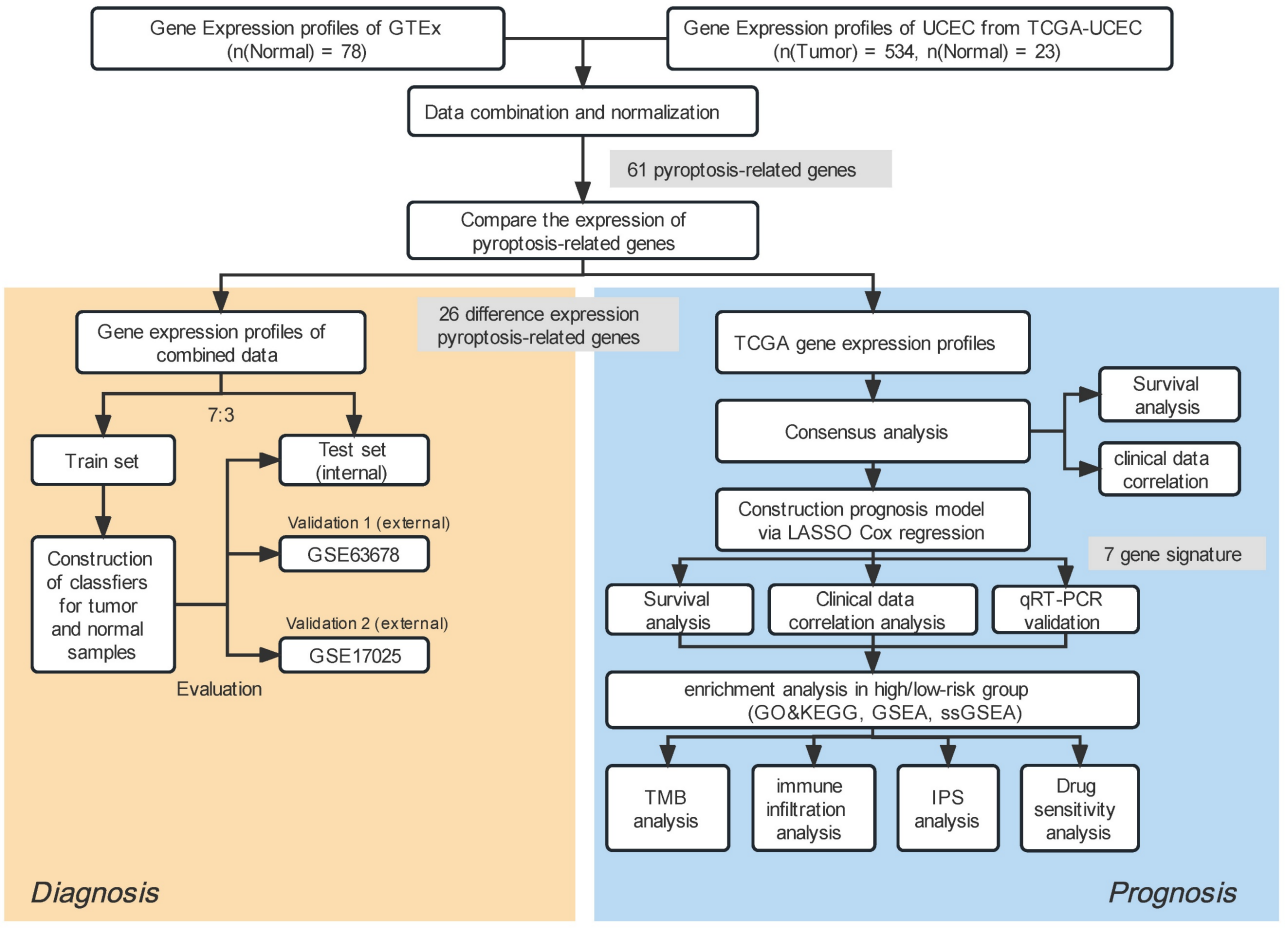
Study workflow.

R (version 4.0.2) and the corresponding package were used in our study to perform all statistical calculations. A statistical differentiation was considered significant if P<0.05 in all two-sided statistical tests. The Student's t-distribution test was applied to juxtapose ordinarily distributed variables across two clusters, whereas the Wilcoxon signed-rank test was adopted to compute continuous variables. The process for our investigation is illustrated in Fig. 1. Others statistical approaches are detailed above.

## 3. Results

### 3.1 DEPRGs identification in normal and UCEC tissues

**Figure 2:**
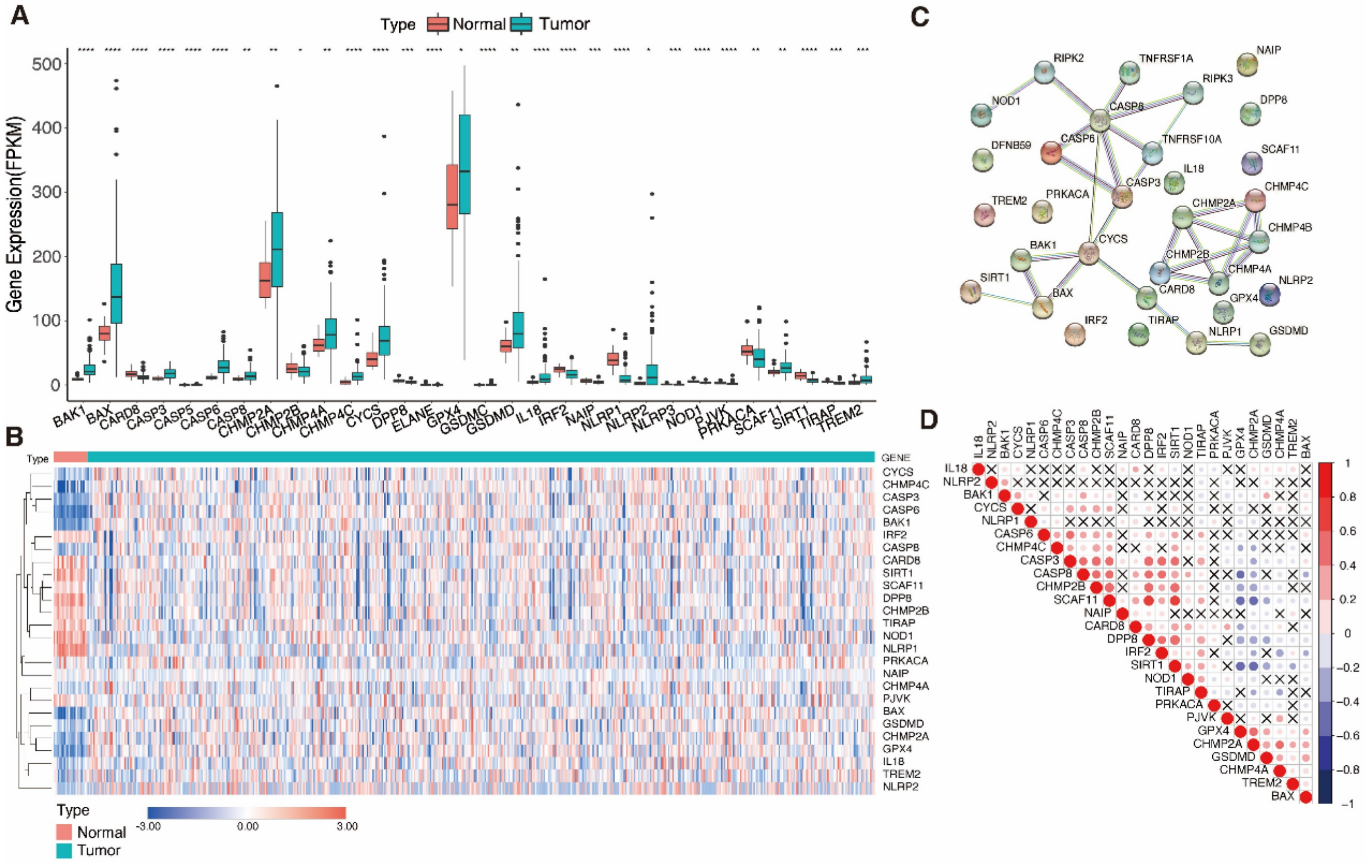
** Identification of differentially expressed pyroptosis-related genes and the associations between them. A** Boxplot of the pyroptosis-related gene (PRG) expression between the normal (red) and tumor (green) samples. P-values are shown as: *P < 0.05, **P < 0.01, ***P < 0.001. **B** Heatmap of the PRG expression between normal samples (red) and tumor samples (blue); blue indicates low expression level and red indicates high expression level. **C** Protein-protein interaction (PPI) network plot indicating the intrinsic association of PRGs (interaction score = 0.9). **D** Heatmap showing correlations between PRGs; blue indicates negative correlation, red indicates positive correlation, and × indicates insignificant correlation).

26 genes had significantly different expression levels when comparing 61 PRGs expression in 534 UCEC samples and 23 normal uterine samples (FDR < 0.05) (Fig. 2A, B). 15 genes (*CASP6, BAK1, CASP3, BAX, CHMP4C, GPX4, CYCS, CASP8, CHMP2A, TREM2, NLRP2, SCAF11, GSDMD, IL18,* and* CHMP4A*) were upregulated and 11 genes (*NLRP1, SIRT1, NOD1, CARD8, DPP8, IRF2, NAIP, TIRAP, PJVK, PRKACA,* and* CHMP2B*) were downregulated in tumor samples. A protein-protein interaction network was built and expression correlation studies were carried out to investigate further the relationships between these DEGs (Fig. 2C, D). For the PPI investigation, a least interaction score of 0.9 was necessary (highest confidence level).

### 3.2 Diagnostic value of DEPRG-based classifiers in UCEC

We next surmised that DEPRGs might be used as markers of UCEC, according to the PRGs expression profile in UCEC. In order to validate this hypothesis, we built diagnostic classifiers using nine standard machine-learning algorithms, including k-NN, LR, SVM, ANN, DT, RF, XGBoost, LightGBM, and CatBoost. We integrated the TCGA-UCEC and GTEx-Uterine datasets. We trained a classifier using the training set RNA-seq data based on the above algorithms. The test dataset was designed to conduct an internal assessment. As expected, the PRG RNA-seq data were suitable for constructing of the UCEC diagnostic classifier because of the high accuracy of the test datasets (Table 1, Table S4). The receiver operating characteristic curves (ROC) performed the classifiers' sensitivity and specificity to assess accuracy. In the testing set, all nine algorithms have AUC values above 0.900 in the test dataset (Table 1).

These observations suggests that PRG can better identify a sample's identity as a tumor or a standard sample. These classifiers' performances on data with varying sample sizes and degrees of proportion were tested employing two external cohorts, and all classifiers performed well according to ROC analysis outcomes.

**Figure 3:**
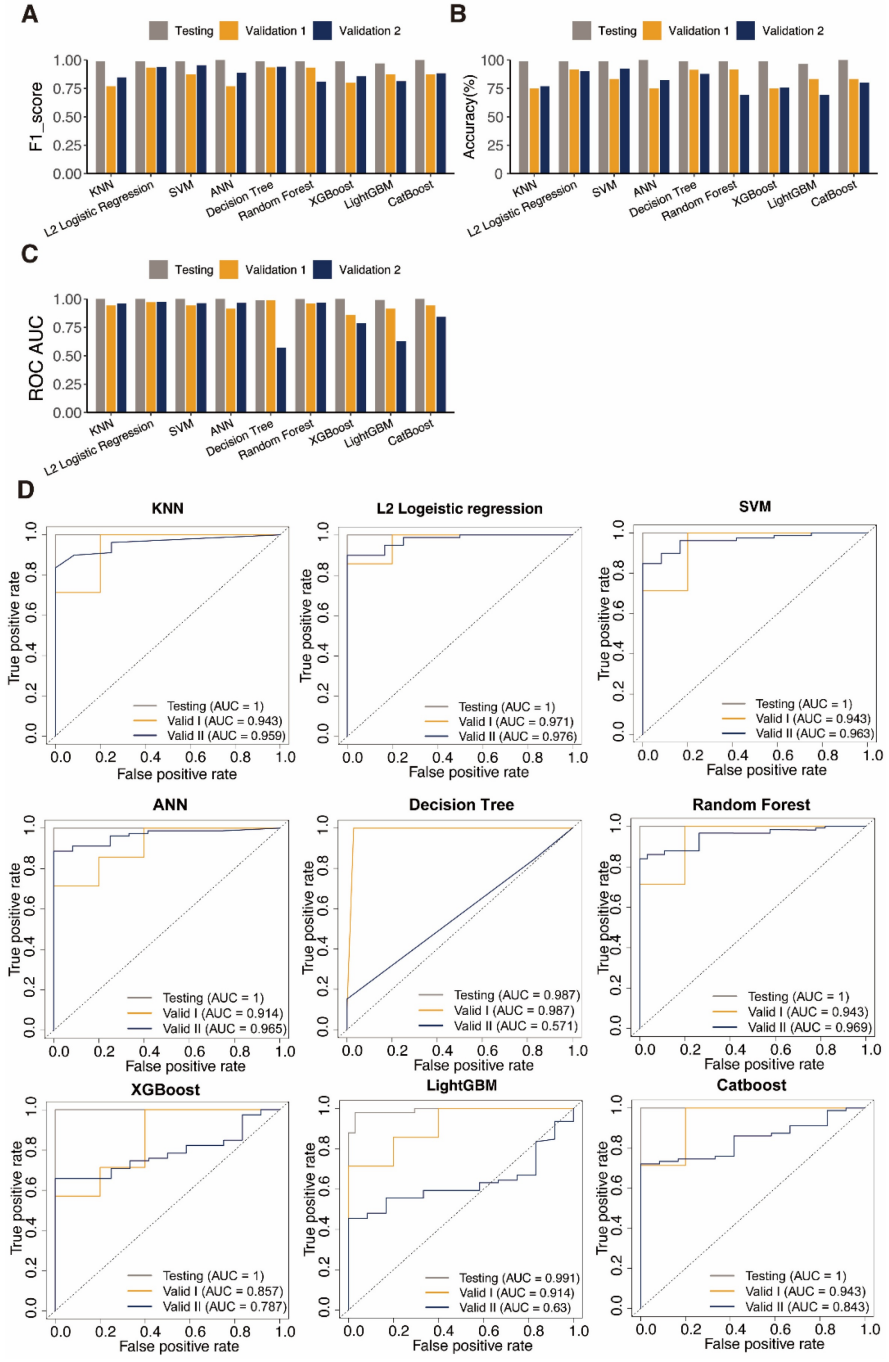
** Performance evaluation of diagnosis classifiers using nine algorithms.** Bars showing the F1 score **A**, the accuracy **B**, and the AUC values **C** of each classifier in the training (orange) and testing (navy) datasets. **D** ROC curves assess the predictive performance of the nine diagnostic models respectively.

The classifiers' accuracy, recall, and F1-score were computed to evaluate them further. The outcomes paralleled those of the ROC analysis. The classifier often has good discriminating power when these values are near 1.000. Furthermore, the classifier is reliable when there is a slight variation in these parameters between datasets. ANN and SVM performed well, indicating that DEPRGs are highly valuable in diagnosing UCEC (Fig. 3). L2 logistic regression was discovered to be the optimal algorithm for creating PRG-based diagnostic models in this study after parameter assessment.

### 3.3 Identification of UCEC clusters

**Figure 4:**
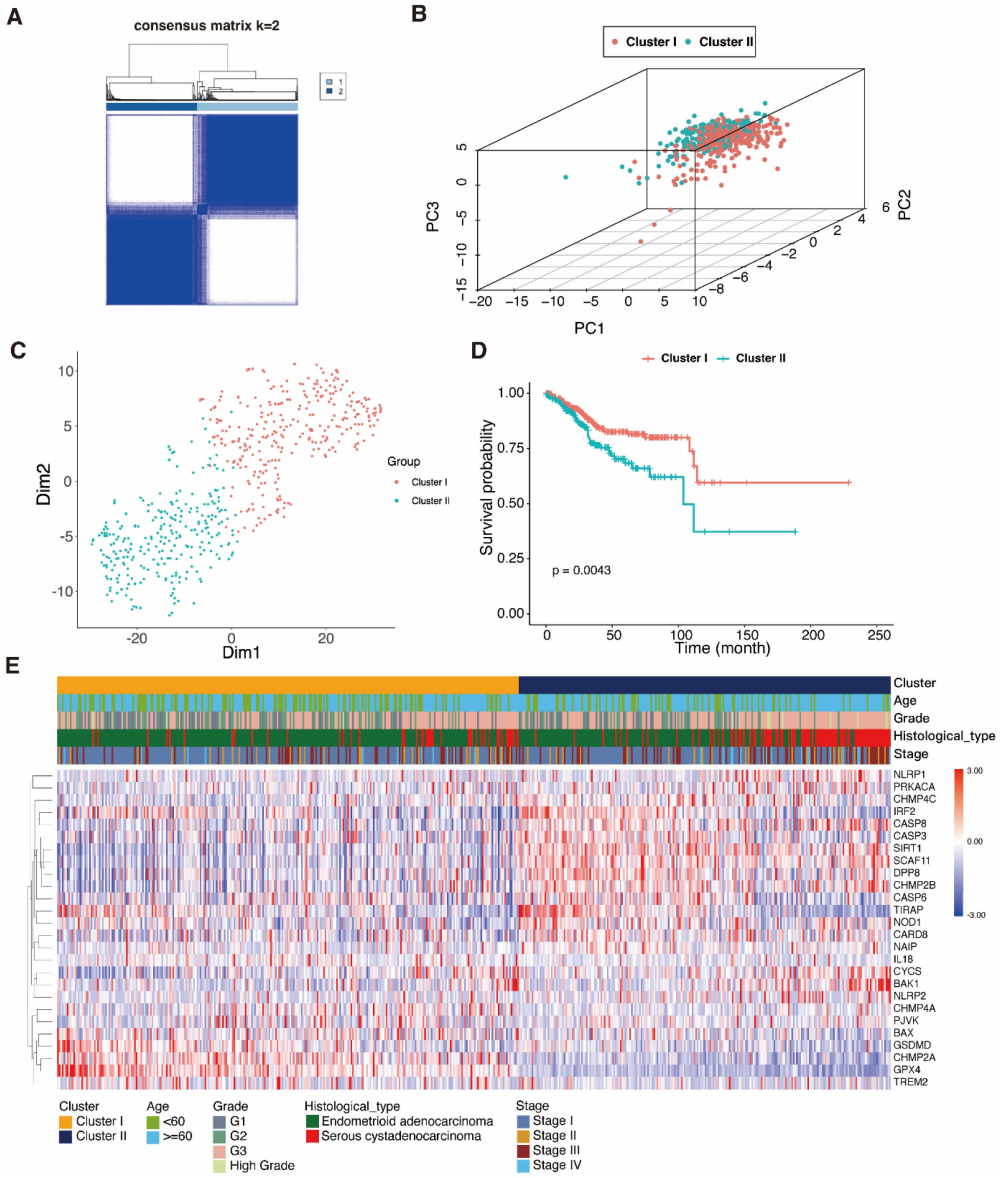
** Consensus clustering analysis of pyroptosis-related genes. A** 534 UCEC samples were grouped into two classes (k = 2), based on the consensus clustering matrix. **B** PCA dot plots represent the class clusters. **C** t-SNE plots represent the two clusters. **D** K-M curves represent the survival times of the two clusters; cluster 1 (red); cluster 2 (blue-green). **E** Heatmap of the two clusters and the UCEC patient clinical characteristics.

In order to examine therapy effectiveness, we explored the connection between DEPRGs expression and UCEC subtypes. Hence, using the DEPRG expression profile as our basis, we did consensus clustering analysis on UCEC patients. The ideal k value for the sample distribution was obtained by varing the categorization value (k) from two to ten. This altered the empirical CDFs until values with the highest levels of stability were found. When k = 2, the expression patterns of the 534 UCEC patient samples (based on the expression of 26 PRGs) could successfully be split into two clusters (Fig. 4A), and this was validated by the PCA and t-SNE (Fig. 4B, C). A heatmap was used to depict the gene expression landscape and patient clinicopathological features, such as the tumor grade, age, and histological type. We observed differences between the two clusters, which were associated with survival time and different stages of UCEC. As shown in the K-M curves, cases in cluster 1 had a greater survival rate compared to those in cluster 2 (Fig. 4D, E).

### 3.4 Key gene identification and evaluation of DEPRG prognostic value in UCEC

**Figure 5:**
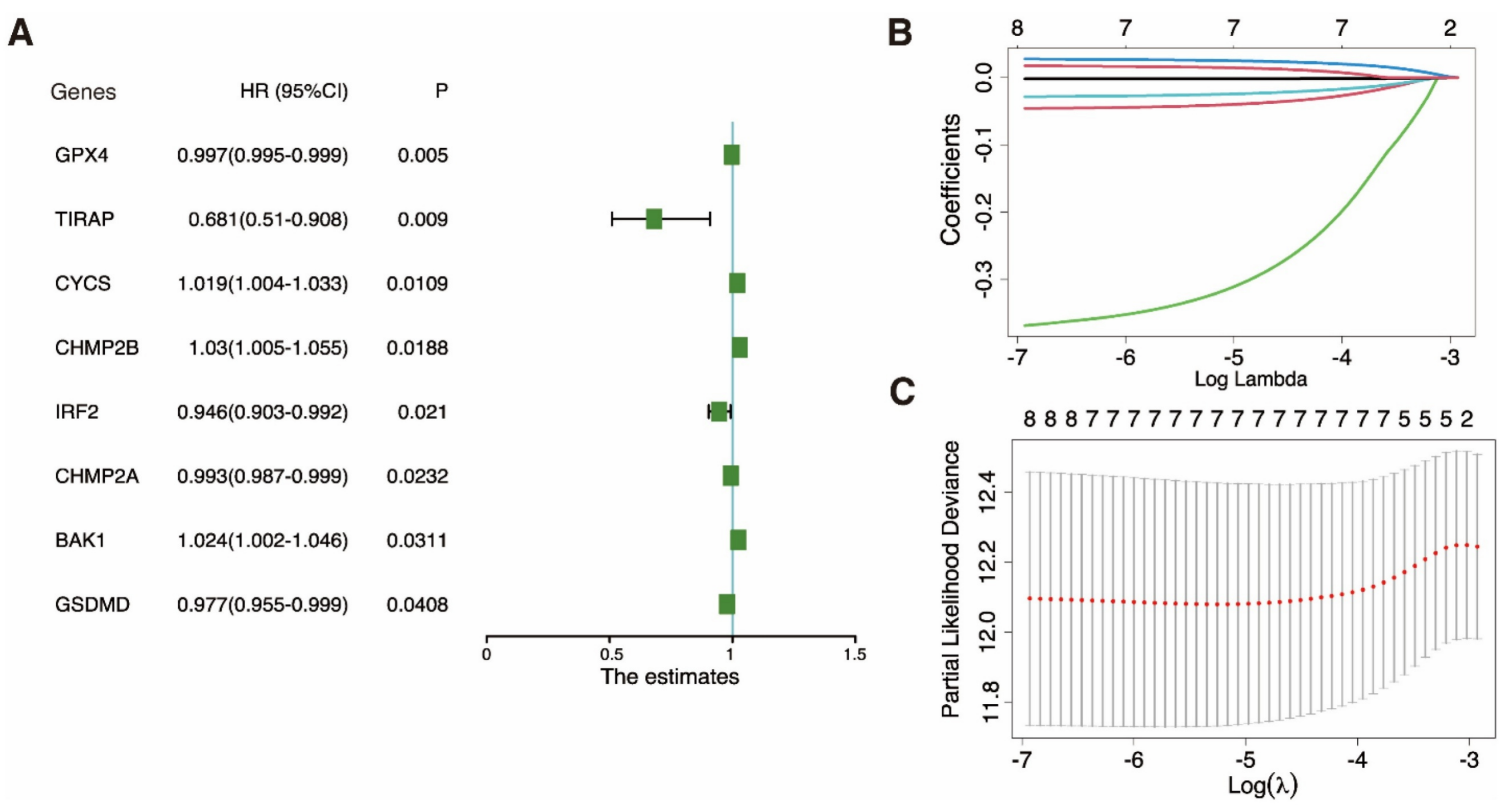
** Construction of a prognostic model based on pyroptosis-related genes. A** Univariate cox regression analysis of individual pyroptosis-related genes (PRGs) yielded significance (P-value) and hazard ratio (HR; 95% confidence interval [CI]) values. **B** LASSO regression analysis of eight survival-related PRGs. **C** Cross-validation for tuning the parameter in the LASSO regression.

Univariate Cox regression was utilized to examine the DEPRGs and assess the prognostic performance. Eight genes (*GPX4, TIRAP, CYCS, CHMP2B, IRF2, CHMP2A, BAK1*, and *GSDMD*) met the P < 0.05 threshold for further analysis. Three (*CYCS, CHMP2B*, and *BAK1*) were risk factors with hazard ratios (HRs) > 1, while the remaining five (*GPX4, TIRAP, IRF2, CHMP2A,* and* GSDMD*) were protective factors among eight genes (Fig. 5A). A LASSO Cox regression framework was adopted for the training dataset in order to minimize candidate genes. Based on the optimal λ, a 7-gene expression prognosis model was built (Fig. 5B, C).

**Figure 6:**
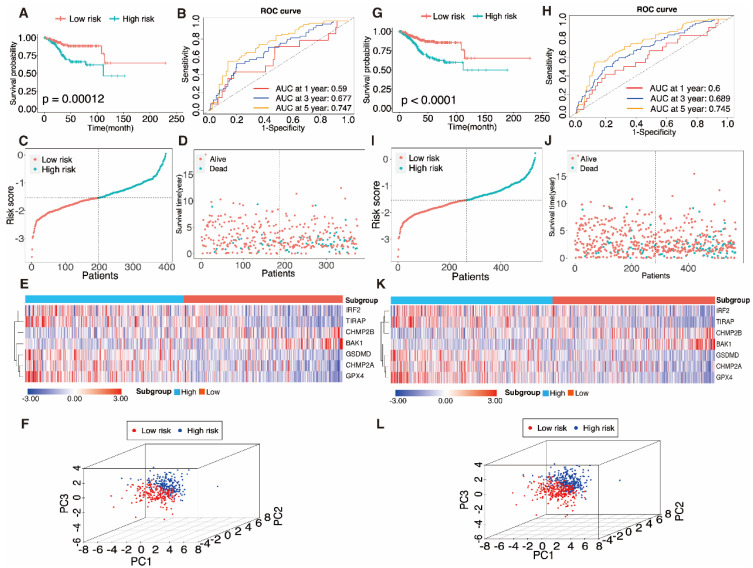
** Evaluation of the pyroptosis-associated gene signature model in the training dataset A-F and the entire dataset G-L. A, G** Kaplan-Meier curves were used to analyze the overall survival (OS) of UCEC patients in the low-risk and high-risk groups. **B, K** Time-determined ROC analysis. Distribution of risk scores **C, I,** survival status D, J, and gene expression E, K in samples from UCEC patients in the low- and high-risk groups according to the risk scores. **F, L** Principal component analysis (PCA).

The aforementioned method was adopted to calculate the risk score in the training set. According to relative coefficients, the seven genes were weighted, and the following calculations were made: Risk score = (-0.041 × *IRF2*) + (-0.326 × *TIRAP*) + (0.026 × *BAK1*) + (-0.025 × *GSDMD*) + (-0.0014 × *CHMP2A*) + (-0.0018 × *GPX4*) + (0.015 × *CHMP2B*). The training dataset (n = 373) was divided into a high-risk sub-category (n = 187) and a low-risk sub-category (n = 186) compared with the median sample. A comparison of the findings displayed a significant distinction in survival condition across the different subgroups (P = 0.00012, log-rank test) (Fig. 6A). The AUC data of PRGRS for the first, third, and fifth-year overall survival had been 0.59, 0.677, and 0.747 (Fig. 6B). Fig. 6C depicts the distribution of the risk scores that we assigned to the UCEC patients. The dot plot displays UCEC patients' residences (Fig. 6D). The heatmap compares the gene expression patterns between the two UCEC patient groups with various prognoses (Fig. 6E), and PCA revealed substantial differences in the two groups of cases (Fig. 6F).

### 3.5 Verifying the predictive capability of PRGRS

To verify PRGRS's prediction performance, the entire dataset was employed. The whole UCEC patient pool was separated into two categories in accordance with the median risk score (i.e., 267 cases scored higher, and 267 cases scored lower). The Kaplan-Meier survival curves of both subgroups were statistically significantly differed from one another (Fig. 6G, P < 0.0001). In the entire dataset, the AUC data had been 0.6, 0.689, and 0.745 for the first, third, and fifth- year overall survival (Fig. 6H). Figure 6I-K depicts the risk scores, case survival state, and PRGRS expression arranged throughout the entire dataset. PCA also showed differences between the groups (Fig. 6L).

### 3.6 PRGRS can independently affect prognosis

**Figure 7:**
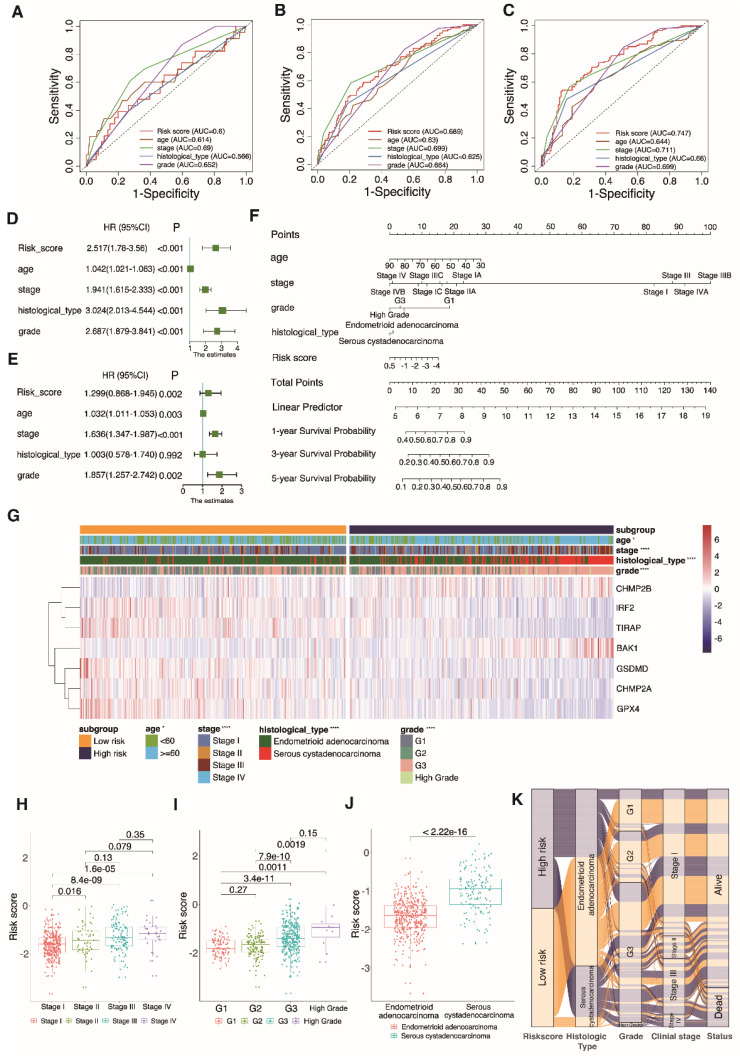
** Selection of independent prognostic factors.** 1-year **A**, 3-year **B**, and 5-year **C** AUCs for clinical factors and risk scores. Univariate and multivariate Cox regression analyses for the validation **D, E** groups. **F** Nomogram predicting the overall survival of UCEC patients at 1, 3, and 5 years by risk score and clinical parameters. **G** Heatmap of clinical parameters for UCEC patients in the low-risk and high-risk groups (*P < 0.05, ****P < 0.0001). **H-J** Boxplots show the differences in risk scores across clinical characteristics. **K** Alluvial diagram establishing associations among risk groups, histological types, grades, clinical stages and survival outcomes.

The predictive strength of the clinical variables and PRGRS was determined by AUC, which is proportional to the signature precision. The risk score computed by PRGRS and the four clinical features is displayed in Fig. 7A-C. The risk score as well as clinical factors (age, stage, grade, and histological type) had been closely related to projection in the univariate Cox analysis (Fig. 7D and Figure S1a). The risk model could independently impact overall survival by adopting multivariate Cox analysis (Fig. 7E and Figure S1b). We created a nomogram figure to display the overall survival rate in UCEC patients in the first, third, and fifth years by combining the prediction framework with a variety of clinicopathological characteristics. Calibration curves were then applied to confirm the nomogram's accuracy (Fig. 7F and Figure S1c). The correlation between PRGRS and clinicopathological features (age, grade, tumor stage, and histological type) is shown in the composite heatmap (Figure 7G). As the risk score rises, the association between risk score and tumor grade becomes more substantial. With a growing tumor grade from grade 1 (G1) to high grade, the boxplot significantly rises in the risk score (P = 0.0011; Fig. 7H). Also, as a patient advanced from stage I to stage IV, the risk score climbed dramatically (P = 1.6 × 10^-5^) (Fig. 7I). Serous cystadenocarcinoma patients scored higher than endometrioid adenocarcinoma patients (Fig. 7J). According to the alluvial diagram, the high-risk group mostly consists of higher-grade tumor subtypes, later clinical stages, and the serous cystadenocarcinoma group, all of which are linked to the poor prognosis (Fig. 7K). Analysis was done on the variations in a number of clinical and pathological features amidst the high and low-risk cohorts (concerning overall survival, Figure S2). The subgroup with low-risk score displayed a superior rate of survival in comparison to the high-risk cohort.

### 3.7 PRGRS expression validation

Compared to normal human endometriosis cells, the expression of *BAK1*, *CHMP2A*, *GPX4*, and *GSDMD* (Figure S3a-d) was upregulated, whereas the expression of *CHMP2B*, *IRF2*, and *TIRAP* (Figure S3e-g) was negatively regulated in UCEC cells. These were in line with the above outcomes.

### 3.8 Analyses of functional and immunological activity

**Figure 8:**
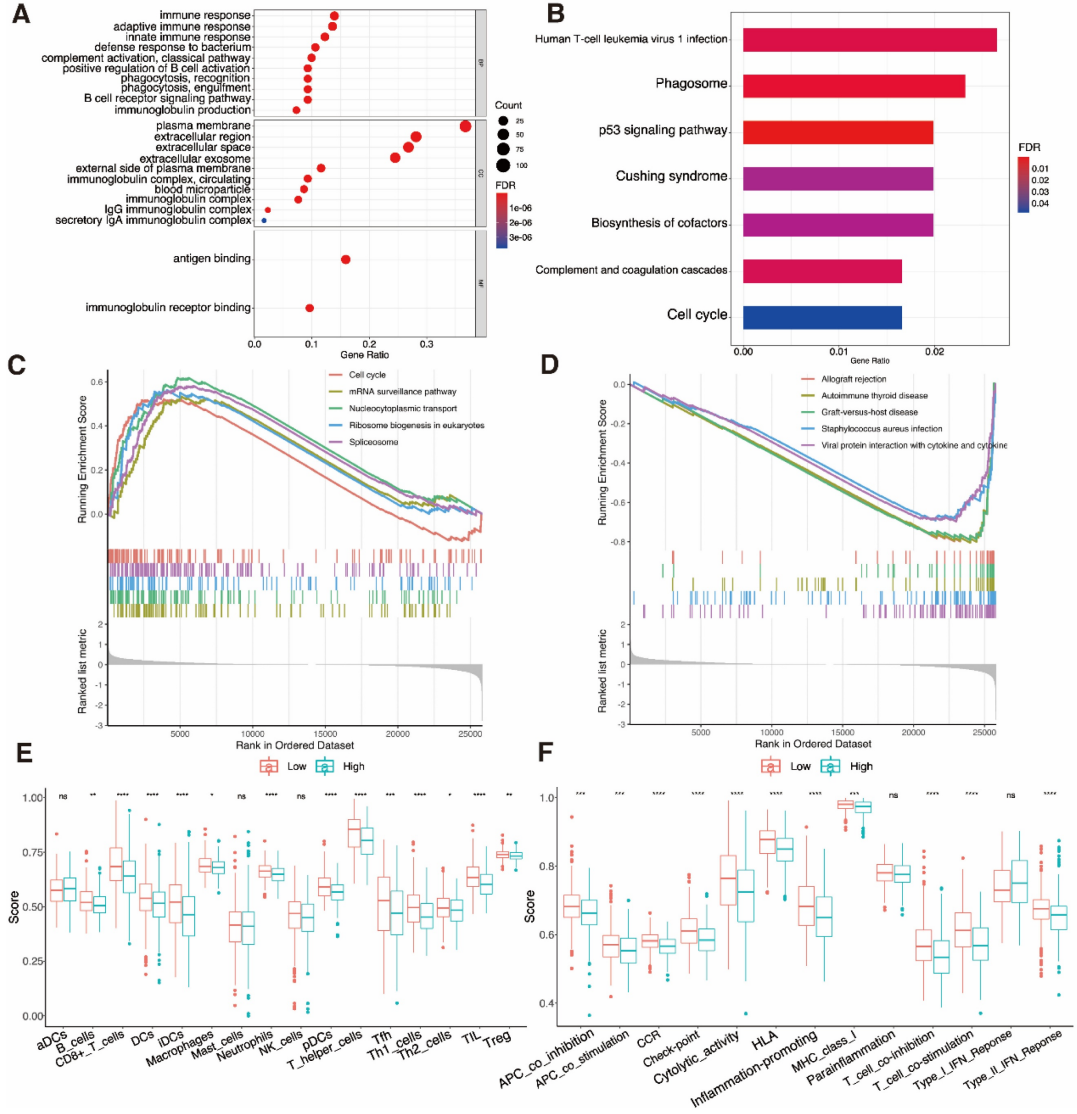
** Functional enrichment analysis. A** Gene ontology (GO) functional enrichment analysis for the differentially expressed genes (DEGs); the count indicates the number of DEGs and the false discovery rate (FDR) indicates the adjusted P-value < 0.05).** B** KEGG enrichment analysis of markers (abscissa: gene percentage of DEGs, FDR: adjusted P-value < 0.05). **C, D** Gene set enrichment analysis based on KEGG of high- and low-risk groups. **E** Comparison of enrichment fractions relating to the 16 immune cell types, between the low-risk (red) and high-risk (green) groups. **F** Comparison of enrichment fractions associated with the 13 immune-related pathways between different low-risk (red) and high-risk (green) groups; ns: not significant, *P < 0.05, **P < 0.01, ***P < 0.001, ****P < 0.0001).

The DEGs sorted from two UCEC subgroups were analyzed by GO and KEGG methods. The outcome of GO functional enrichment depicted the DEGs as primarily enriched in immune-related processes, such as the B cell receptor signaling pathway and complement activation (Fig. 8A). The KEGG enrichment results indicated that the DEGs mainly engage in human T cell leukemia virus 1 infection, and the phagosome and p53 signaling pathways (Fig. 8B). We discovered through GSEA that the high-risk subgroup is connected to pathways that are related to tumors (Fig. 8C). Low-risk subgroup cases have immune-related pathways (Fig. 8D). The index associated with immune cells and immune-related pathways in two risk subgroups was further analyzed by ssGSEA. We discovered that PRGRS and immunogenicity had an opposite relationship. The index low-risk cluster exhibited an elevated incidence compared to the high-risk category, which includes the majority of immune cells such as B cells, CD8^+^ T cells, dendritic cells (DCs), interstitial (i)DCs, plasmacytoid (p)DCs, macrophages, neutrophils, T helper cells (Th1 and Th2), T follicular helper (Tfh) cells, tumor-infiltrating lymphocytes (TILs), and regulatory T cells (Tregs) (Fig. 8E, P < 0.001). Except for the parainflammation and type I interferon (IFN) responses, the low-risk group has higher activation levels across all eleven immunological pathways than the high-risk cohort (Fig. 8F).

**Figure 9:**
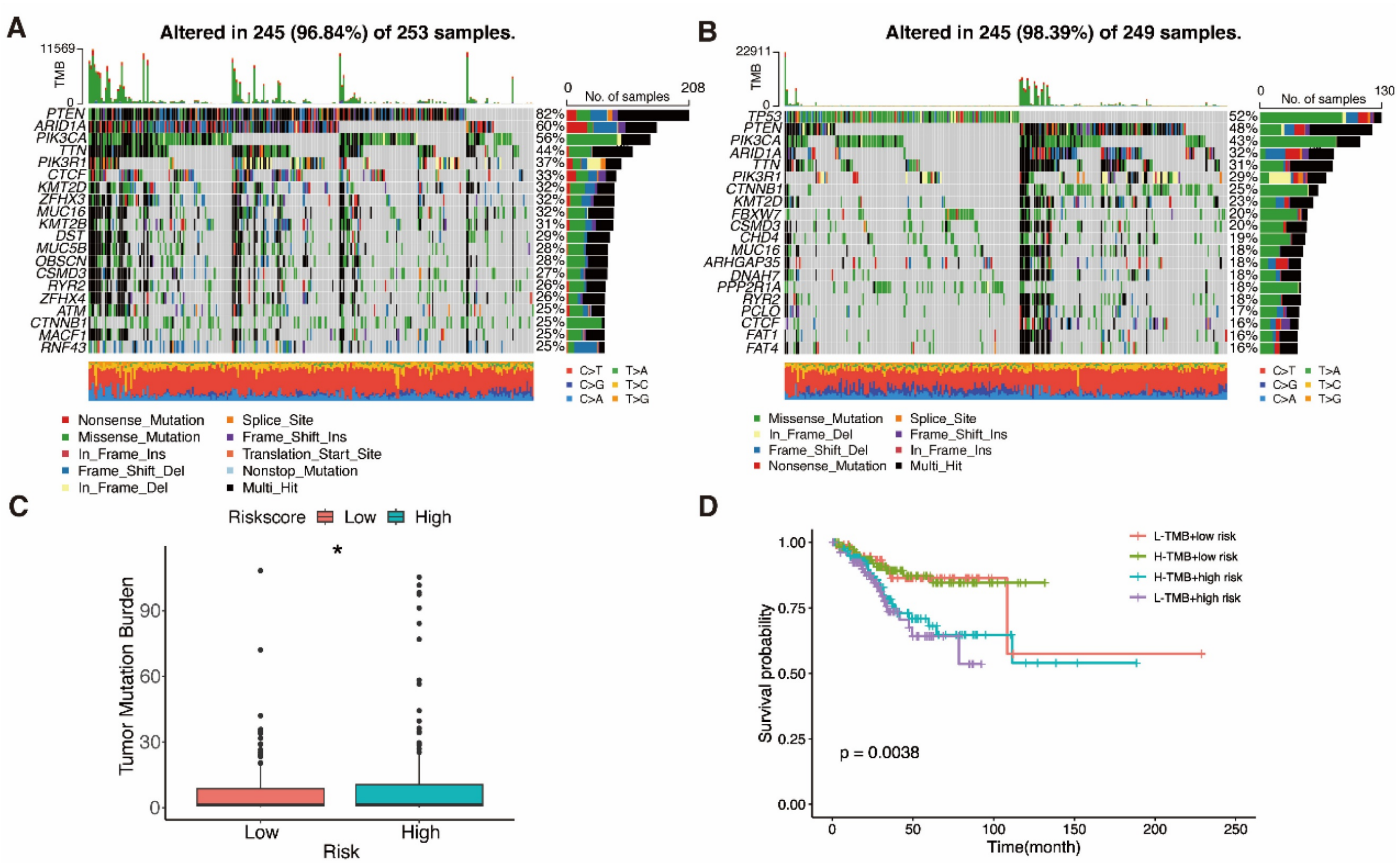
** Mutation analysis. A, B** Waterfall plots show mutation profile of low- and high-risk groups. **C** Boxplot shows the relationship between the risk-score and tumor mutation burden (TMB). **D** K-M curves represent the association of TMB and prognosis in TCGA UCEC dataset.

Finding patients who react appropriately to ICIs may be done using TMB, which has always been a major factor impacting immunotherapy 61. Waterfall plots were used in our study to display high mutation frequency gene mutation information in both subgroups, respectively (Fig. 9A, B). Patients with lower TMB value and high risk had the lowest chances of surviving in four groups, according to the TMB coupled with the risk score survival analysis (Fig. 9D, P = 0.0038). Low-risk subgroup exhibited harder TMB (Fig. 9C, P = 0.023).

### 3.9 Drug sensitivity test screening of six potential chemotherapy drugs

**Figure 10:**
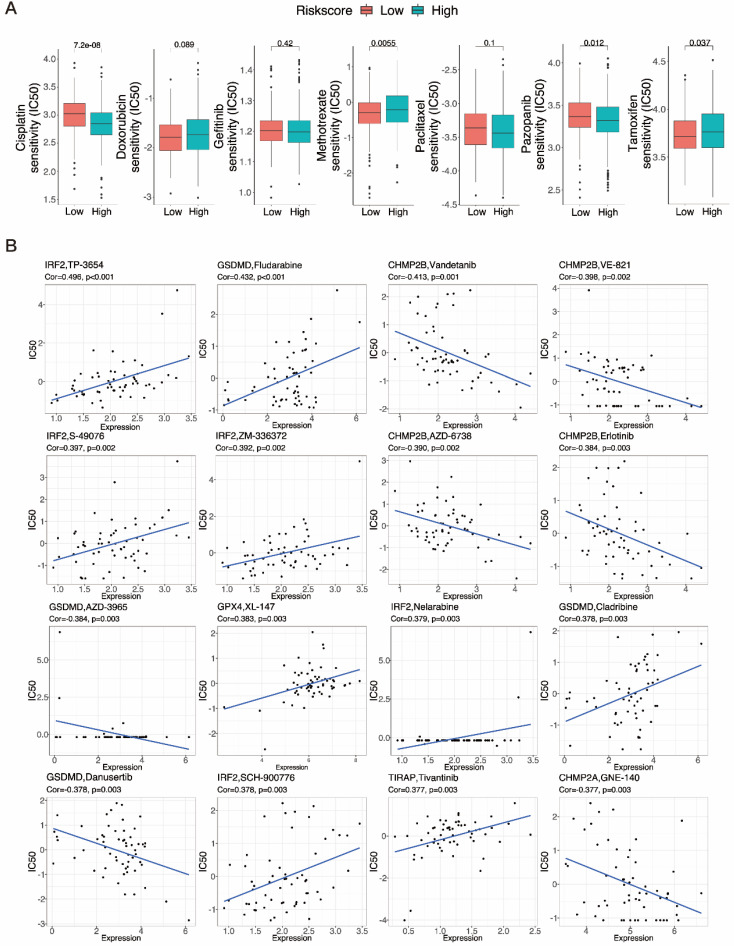
** Results of chemotherapy drug sensitivity analysis. A** The relationship between risk score and chemotherapy drug sensitivity in UCEC. Boxplots show the estimated IC50 values for cisplatin, doxorubicin, gefitinib, methotrexate, paclitaxel, pazopanib and tamoxifen in the low- and high-risk groups. **B** Scatter plots indicate the relationship between prognostic gene expression and drug sensitivity.

Chemotherapy is commonly used to treat patients with UCEC. Hence, we investigated how the two subgroups of UCEC patients responded to seven pharmacotherapeutic agents, comprising cisplatin, doxorubicin, gefitinib, methotrexate, paclitaxel, pazopanib and tamoxifen. We used markers to determine the IC50 for each sample. The results revealed that the efficacy of four of these medications differed significantly between the groups (Fig. 10A). Cisplatin (P < 0.001) and pazopanib (P = 0.012) both exhibited higher IC50s in the low-risk subgroup, indicating that these chemotherapeutic drugs would be more effective in high-risk cases. Methotrexate (P = 0.0055) and tamoxifen (P = 0.037), in contrast, could be more beneficial in the low-risk subgroup. The tiny molecule substances closely related to the expression of PRGRS were then further investigated. The outcomes revealed all six genes were strongly related to sensitivity to various compounds (P < 0.01) (Fig. 10B, Table S6). Increased expression of *IRF2*, *GPX4*, and *TIRAP*, for example, has been linked to enhanced susceptibility of tumor cells to chemotherapeutic agents such as TP-3654, nelarabine, S-49076, ZM-336372, XL-147T, and tivantinib. In addition, the expression of *CHMP2B* and *CHMP2A* was associated with increased resistance to vandetanib, VE-821, erlotinib, AZD-6738, and GNE-140. Moreover, Fludarabine and cladribine resistance were positively connected with *GSDMD* expression, while AZD-3965 and danusertib resistance were negatively correlated. These mechanisms need to be further investigated.

Also, the boxplot depicted that the PRGRS gene expression varied significantly in two subgroups (Figure S4a). A substantial correlation between survival rate and gene expression was displayed by the K-M curves for individual genes in PRGRS (Figure S4b).

### 3.10 Immune infiltration and PRGRS provide a significant link

**Figure 11:**
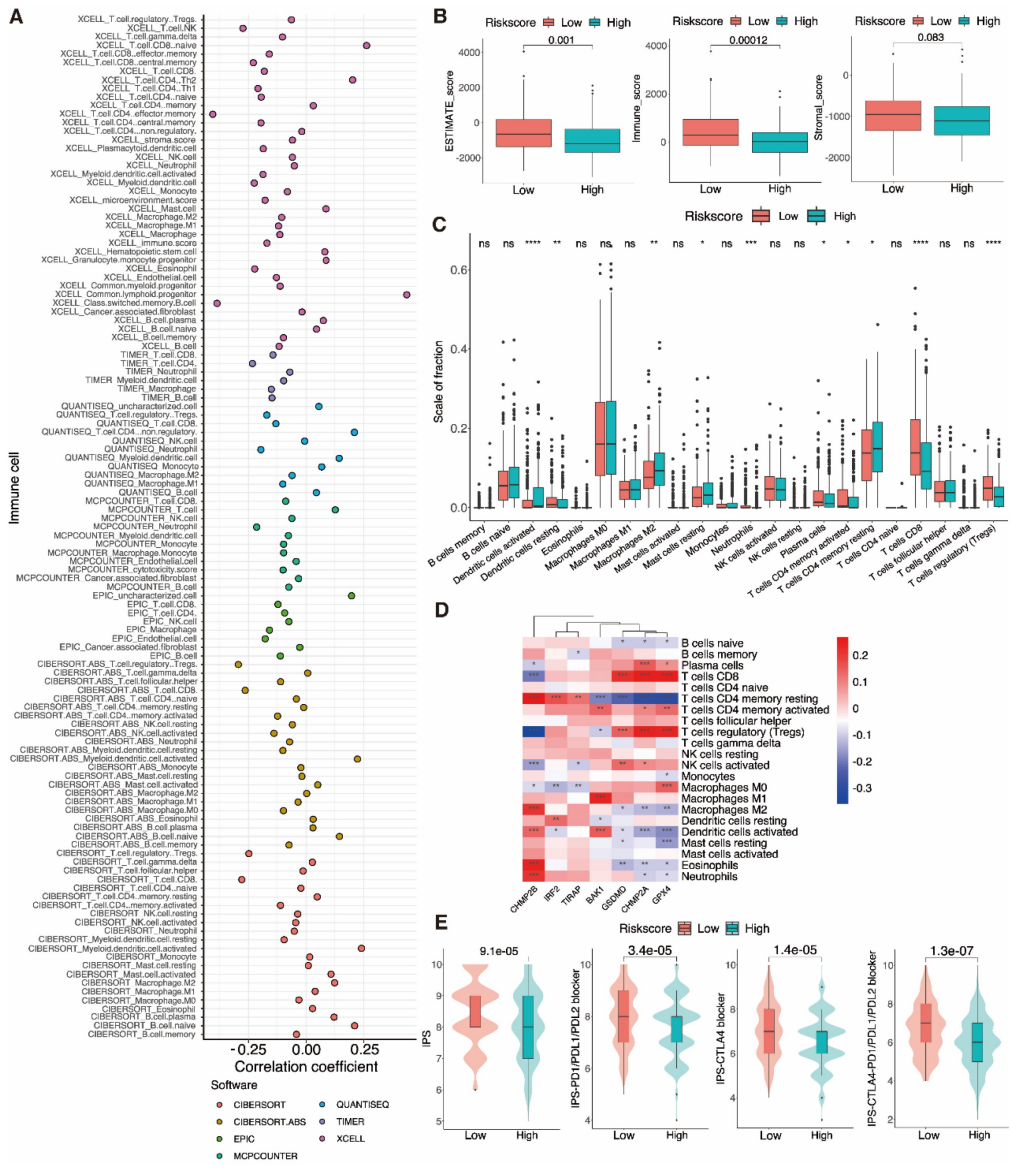
** Immuno-correlation analysis of tumor infiltration. A** Multiple analyses of correlation coefficients between different immune cells and risk scores. **B** Boxplots show the differences in the ESTIMATE, immune, and stromal scores between the low-and high-risk groups of UCEC patients, calculated using the ESTIMATE algorithm. **C** The extent of immune cell infiltration in the low- and high-risk groups of UCEC patients. **D** A heatmap showing correlations between the expression of signature genes and the abundance of immune cells. **E** Boxplot showing IPS scores for different risk groups at various immune checkpoints.

After discovering a negative association between immunological infiltration and PRGRS, platforms such as ESTIMATE, CIBERSORT, EPIC, XCELL, etc. were adopted to assess any association between PRGRS and immune infiltration (Fig. 11A). The study of the differences in ESTIMATE, immunological, and stromal scores among the low and high-risk sub-categories was done via the ESTIMATE method. The findings indicated that immunogenicity and risk scores were inversely correlated (Fig. 11B). We thoroughly assessed the quantity of 22 immune cell species in the two sub-categories using CIBERSORT (Fig. 11C and Figure S5a). For low-risk UCEC cases opposite to the high-risk, the abundance of resting DCs, neutrophils, plasma cells, activated memory CD4+ T cells, CD8+ T cells, and Tregs was significantly larger. In contrast, there were proportionately more resting memory CD4+ T cells, M2 macrophages, resting mast cells, and activated DCs of the high-risk subgroup than the other. We next evaluated the associations linking the PRGRS expression and the distribution of immune cells within both subgroups of UCEC samples (Fig. 11D and Figure S5b). We discovered the immune cells' filtration was impacted by PRGRS expression. The connection between the IPS and PRGRS was further examined. The low-risk subgroup cases scored on the IPS (IPS-CTLA-4, IPS-CTLA-4/PD-L1/PD-L1, and IPS-PD-1/PD-L1/PD-L2) far better greater than those in the high-risk. IPS was adopted to determine the likelihood for responding to ICI therapy (Fig. 11E). We conclude that low-risk patients were more likely to mount an immunological response following ICI treatment.

## 4. Discussion

UCEC is a type of prevalent gynecological cancer 67. Patients with UCEC are typically treated using surgery, postoperative radiation, and chemotherapy, which need to be tailored based on the type or stage of the tumor 68. Pyroptosis, an identified pattern of programmed cell death, has both pro- and anti-cancer functions. For instance, pyroptosis stimulates the production of inflammatory factors, which help transform normal cells into tumor cells. Conversely, various therapeutic strategies aim to induce tumor cell pyroptosis 69. Diagnostic and prognostic models are valuable tools for demonstrating the importance of pyroptosis in cancer. In this study, 26 of the 61 PRGs were differentially expressed in UCEC versus normal uterine samples. Based on the differential expression of these PRGs, diagnostic classifiers were created by applying machine learning algorithm to evaluate the diagnostic utility of PRGs in UCEC. The differential gene consensus clustering analysis results showed differences in prognosis between the two clusters of UCEC cases. Then, to examine the prognostic capacity of UCEC, we created a seven-gene risk model using LASSO Cox regression. The DEGs (between the low- and high-risk groupings) had been connected to processes that are relevant to immunity, according to functional enrichment analysis. The PRGRS might be utilized to forecast the immune cell content and drug sensitivity of UCEC tumors. It is thus anticipated that this signature will support UCEC immunotherapy, diagnosis, and prognosis.

Nowadays, non-invasive tests are not available, and there is a lack of early screening for asymptomatic or high-risk populations. We constructed classifiers to assess differences between tumor or normal tissue, using nine well known machine learning algorithms. The Classifiers based on logistic regression, SVM, and ANN performed particularly well. Other than the decision tree, classifiers created utilizing tree-based algorithms such as random forest, XGBoost, LightGBM, and CatBoost performed well. The unsatisfactory classification of some models may be because the model's various parameters need further detailed optimization. Tumor heterogeneity may have contributed to the diagnostic models' inconsistent performance in validation sets 1 and 2; these models fared well despite that. Therefore, additional training samples must be gathered, and the parameters must be fine-tuned to further optimize the diagnostic model for UCEC. For robustness validation, additional datasets are still required. The above findings imply that the PRG-based signature may distinguish EC cells from normal cells and initial screening for cancer during the patient's physical examination. For patients classified as tumors by the model, the diagnosis is further confirmed by clinical examination so that treatment strategies can be determined earlier.

Once we had demonstrated that the expression profiles of PRGs had prognostic value for UCEC, we set out to verify this hypothesis using UCEC patient data. We found that patients in two clusters separated by PRGs have different prognoses. This shows the prognosis of UCEC patients may vary depending on the occurrence of pyroptosis in the tumor. Then, using LASSO Cox regression analysis, we built a prognostic signature corresponding to seven PRGs, with patients in various risk subgroups having various overall survival rates. These observations show that the prognosis of UCEC patients may vary depending on pyroptosis in the tumor.

PRGRS is an autonomous predictive variable for UCEC via univariate and multivariate regression examinations. The tumor grade, histological type, and cancer stage exhibited substantial differences between the two risk clusters while comparing the patient clinical features. The serous cystadenocarcinoma group, higher-grade tumor subtypes, and later clinical stages—all of which are linked to a poor prognosis—are mostly represented in the high-risk subgroup, further validating the reliability of our model in assessing the severity of patients' conditions 70,71. The model's strong predictive performance was supported by the AUC values. The resulting nomogram also showed that PRGRS could accurately predict the prognosis of UCEC individuals 53. When compared to prior research, the PRGRS has superior prediction performance for patients at 3 years (AUC=0.689 > 0.682, 0.662, 0.658) and 5 years (AUC=0.745 > 0.723, 0.717, 0.659), indicating that the model may be able to discern between the severity of patients' conditions early for better treatment 72-74. Meanwhile, the genes utilized to build the model in this work were preliminary validated for the accuracy of gene expression changes using qRT-PCR experiment.

During the screening of seven PRGs, *IRF2* edits keratinocytes exhibit enhanced clonogenic and migratory characteristics. We hypothesize that it has a driving effect on tumor development and migration. *IRF2* appeared as a vital modulator of the inflammatory vesicles' stimulation in human macrophages and endothelial cells, by inducing an inflammatory vesicle-mediated response. *IRF2* is also crucial for the expression of *GSDMD*, which is directly implicated in pyroptosis induction 75-77. *GSDMD* is activated by the cleavage of inflammatory cysteases such as caspase-1/4 or neutrophil elastase, which leads to pore formation, pyroptosis, and cytokine release 8. *GSDMD* activation is also significantly associated with the induction of neuronal pyroptosis, which occurs following stroke 78. In addition, *TIRAP* participates in immune signal transduction, controls caspase-11 production during pyroptosis, and is crucial for inflammatory vesicle activation 79,80. *GPX4* activity is crucial for regulating lipid homeostasis in cells to avoid the buildup of harmful lipid reactive oxygen species (ROS), which stops the ferroptosis oxidative, iron-dependent, non-apoptotic processes of cell death 81. Several studies have also demonstrated its role in tumor therapy 82-84. The pro-apoptotic protein *BAK1* has been identified as a prognosis marker for advanced gastric cancer after treatment 85. By interacting with miRNA-125 family members, *BAK1* forms part of a regulatory pathway related to tumor growth 86,87. Two ESCRT-III isoforms, *CHMP2A* and *CHMP2B*, have different membrane-associated properties and are engaged in a variety of membrane remodeling procedures. During pyroptosis, cell membrane damage and repair are mediated by the ESCRT machinery 88,89. However, there are no reports of the roles of *CHMP2A* and *CHMP2B* in tumors. Our findings suggest that a worse prognosis may be associated with a lower degree of *CHMP2A* expression. Thus, prompting the upregulation of *CHMP2A* may be a plausible therapeutic strategy.

Based on GO and KEGG enrichment outcomes, immune response regulation, complement system activation, and the p53 signaling pathway were all associated with the expression of PRGRS grouped using the model. GSEA outcomes illustrated that the high-risk subgroup is connected to pathways that are related to tumor development. Immunoreactive pathways are amplified in samples where the risk is low. These findings demonstrate that PRGRS is related to immunity. Meanwhile, ssGSEA and ESTIMATE showed that immune cell infiltration into the tumor was often lower in high-risk individuals than in low-risk UCEC individuals. Moreover, The high-risk subgroup's ESTIMATE, immunological, and stromal scores were lower than those in the other, which is consistent with other research 67. These findings suggest that all UCEC individuals may gain advantages from immunotherapy as a whole.

Responses of UCEC patients were then evaluated to numerous popular chemotherapy treatments. It was discovered that high-risk cases responded better to cisplatin and pazopanib than low-risk patients did, implying that the high-risk subgroup benefited better from these chemotherapeutic drugs. Yet, methotrexate and tamoxifen were more readily reacted to by patients with low scores. These findings could be used to select appropriate chemotherapy regimens for UCEC patients in different risk categories.

Immune cell infiltration is related to clinical prognosis in individuals with UCEC. The abundance of various tumor-infiltrating cells varied across the two subgroups. Quiescent DCs, neutrophils, plasma cells, memory CD4^+^ T cells, memory CD8^+^ T cells, and Tregs were more prevalent in the tumors of lower-risk UCEC patients. By contrast, high-risk individuals had more significant proportions of tumor-infiltrating resting memory CD4^+^ T cells, resting mast cells, and M2 macrophages. TAMs are M2-polarized macrophages that encourage the growth and dissemination of tumor cells and block T-cell-regulated anti-tumor immune responses, hence accelerating tumor development 90. Yang J. et al. 91 found CD8^+^ T cells, neutrophils, and Tregs were linked to the UCEC patients' survival. Moreover, Chen B. et al. 92 confirmed that the degree of Treg infiltration correlated favorably with the survival rates of UCEC individuals. We discovered important immune infiltrating cells that varied between groups, suggesting possible targets for further research. Given these results, more studies are needed to determine the link between pyroptosis, immune cell infiltration, and cases' prognosis.

We also observed that the immune checkpoint molecules CTLA-4, PD-1, and PD-L1 were substantially further conveyed in the low-risk cases than the others. Many malignancies have been successfully combated by antibodies that target CTLA-4, PD-1, and PD-L1 93. In addition, inhibitors of PD-1 and PD-L1 are being utilized successfully as immunotherapies for several recurring or metastatic malignancies 94. Patients with UCEC respond well to PD-1-targeting ICIs 95. Jiang Y. et al 96 discovered that GSDMD was favorably linked with immune checkpoint features and negatively impacted anti-tumor immunity during anti-PD-L1 therapy. TMB was utilized to evaluate signatures' propensity to recognize patients who respond more favorably to ICIs. In UCEC low-risk groups, TMB performed higher. As a result, immunotherapy could be more fruitful for low-risk cases that may be recognized by immune cells. Thus, Patients at low risk may respond better to immunotherapy. Furthermore, immunotherapy can be paired with traditional chemotherapy and surgery for improved patient outcomes.

The use of candidate genes in the present study to build diagnostic and prognostic models demonstrates PRGs utility in predicting UCEC diagnosis and prognosis. To create the PRG-based diagnostic model, we examined the efficacy of several conventional machine-learning algorithms. In future studies, we will continue to analyze the potential of these models to predict UCEC metastasis. We solely used the mRNA levels of protein-coding PRGs to create a prognostic model for UCEC. This model outperformed traditional models based on patient clinical features and was significant in predicting the prognosis of UCEC, while being distinct from some other gene-based models. Our analysis may be constrained by the small sample size because it mainly employed a single TCGA-UCEC dataset. Therefore, our results need to be confirmed using broader datasets and further backed by further clinical and *in vivo* experimental data.

## Conclusions

In this study, using genomic, transcriptomic, and clinical data of endometrial cancer from TCGA and GEO databases, we developed diagnostic models and a prognostic model based on pyroptosis-related genes and discovered the ability of pyroptosis-related genes to classify normal and cancer samples, predict patient prognosis, and associate with immune response. The ability of these DEPRGs to differentiate between normal and UCEC tumor tissues was subsequently proven. Furthermore, employing a prognostic model according to seven PRGs' expression which can independently affect UCEC prognosis, we identified a significant correlation between immune infiltration and prognostic model. Therefore, our research has identified novel genetic signatures of UCEC patients, which could be used in diagnosis and prognosis prediction, as well as the selection of appropriate anti-cancer treatments.

## Supplementary Material

Supplementary figures and tables.

## Figures and Tables

**Table 1:** Evaluation of classifiers generated using different machine learning algorithms.

Classifiers	Dataset	Precision	Recall	F1_score	Accuracy	ROC AUC
KNN	Testing	1.000	0.980	0.990	98.81%	1.000
	Validation 1	0.833	0.714	0.769	75.00%	0.943
	Validation 2	1.000	0.734	0.847	76.92%	0.959
L2 Logistic Regression	Testing	0.980	1.000	0.990	98.81%	1.000
	Validation 1	0.875	1.000	0.933	91.67%	0.971
	Validation 2	1.000	0.886	0.940	90.11%	0.976
SVM	Testing	0.980	1.000	0.990	98.81%	1.000
	Validation 1	0.778	1.000	0.875	83.33%	0.943
	Validation 2	0.974	0.937	0.955	92.31%	0.963
ANN	Testing	1.000	1.000	1.000	100.00%	1.000
	Validation 1	0.833	0.714	0.769	75.00%	0.914
	Validation 2	1.000	0.797	0.887	82.42%	0.965
Decision Tree	Testing	0.980	1.000	0.990	98.81%	0.987
	Validation 1	0.700	1.000	0.824	75.00%	0.987
	Validation 2	0.870	0.848	0.859	75.82%	0.571
Random Forest	Testing	0.980	1.000	0.990	98.81%	0.999
	Validation 1	0.875	1.000	0.933	91.67%	0.943
	Validation 2	0.870	0.759	0.811	69.23%	0.730
XGBoost	Testing	0.980	1.000	0.990	98.81%	1.000
	Validation 1	0.750	0.857	0.800	75.00%	0.857
	Validation 2	0.880	0.835	0.857	75.82%	0.787
LightGBM	Testing	0.961	0.980	0.970	96.43%	0.991
	Validation 1	0.778	1.000	0.875	83.33%	0.914
	Validation 2	0.859	0.772	0.813	69.23%	0.630
CatBoost	Testing	1.000	1.000	1.000	100.00%	1.000
	Validation 1	0.778	1.000	0.875	83.33%	0.943
	Validation 2	0.907	0.861	0.883	80.22%	0.843

## Abbreviations

AUC: Area under the curve
CI: confidence interval
CTLA4: Cytotoxic T-Lymphocyte associated protein
DEPRGs: differential expression pyroptosis-related genes
EC: Endometrial carcinoma
FDR: False discovery rate
GEO: Gene Expression Omnibus
GO: Gene ontology
GSEA: Gene set enrichment analysis
GTEx: Genotype-Tissue Expression
HR: Harzad ratio
ICIs: Immune checkpoint inhibitors
KEGG: Kyoto encyclopedia of genes and genome
LASSO: least absolute shrinkage and selection operator
OS: overall survival
PCA: Principal Component Analysis
PD-1: programmed death-1
PD-L1: Programmed cell death 1 ligand 1
PPI: protein-protein interaction
PRG: Pyroptosis-related gene
PRGRS: Pyroptosis-related gene risk signature
ROC: Receiver operating characteristic
ssGSEA: single sample gene set enrichment analysis
TMB: Tumor Mutation Burden
TME: Tumor microenvironment
t-SNE: t-distributed stochastic neighbor embedding
