# Supplementary material for: Pyroptosis-Related Gene Signatures Enable Robustly Diagnosis, Prognosis and Immune Responses Prediction in Uterine Corpus Endometrial Carcinoma
Source: J Cancer. 2025 May 8;16(8):2516–36. doi: 10.7150/jca.104826 (PMC12171010; doi:10.7150/jca.104826)
Supplement: Supplementary file 1 — Supplementary figures and tables. [file jcav16p2516s1.pdf]

Supplementary files

FIGURES

1. Figure S1: Univariate and multivariate Cox regression analyses for the training (A, B) groups. (C) The calibration curves show the accuracy of the nomogram at 1, 3, and 5 years.

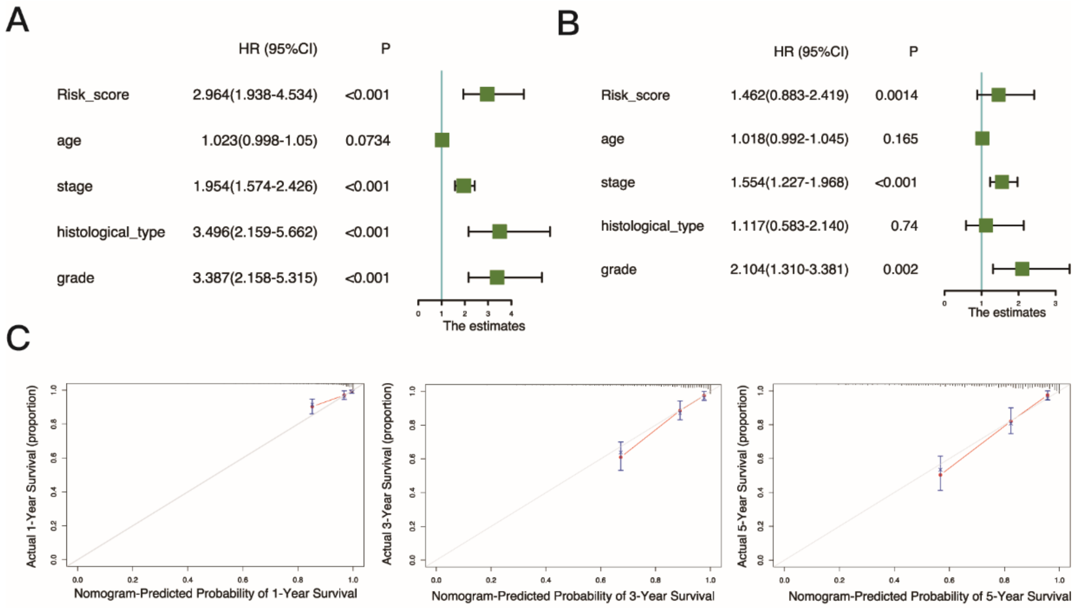

2. **Figure S2**: K-M curve analyse the differences in several clinicopathological features between high- and low-risk groups.

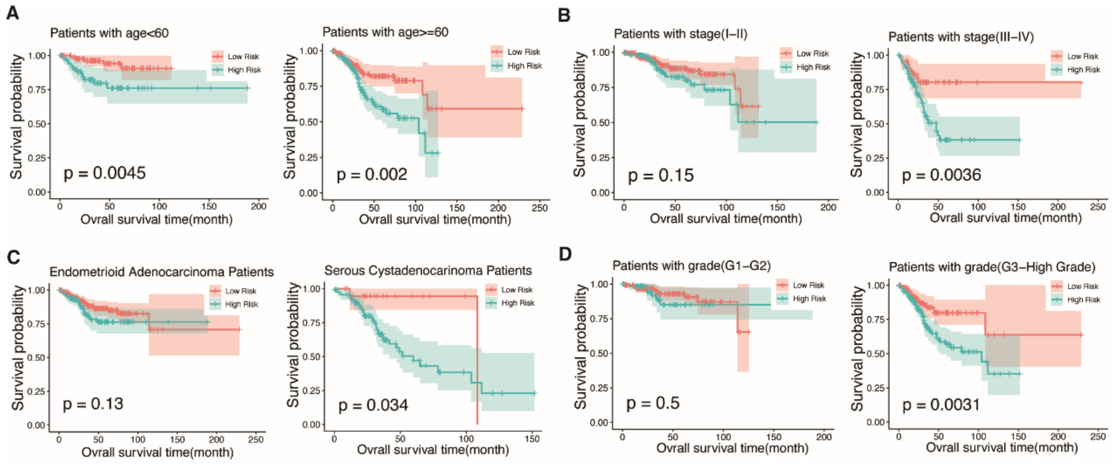

3. **Figure S3:** Boxplots present significant differences in the expression of BAK1 (A), CHMP2A (B), GPX4 (C), GSDMD (D), CHMP2B (E), IRF2 (F), and TIRAP (G) in UCEC cells versus normal human endometriosis cells.

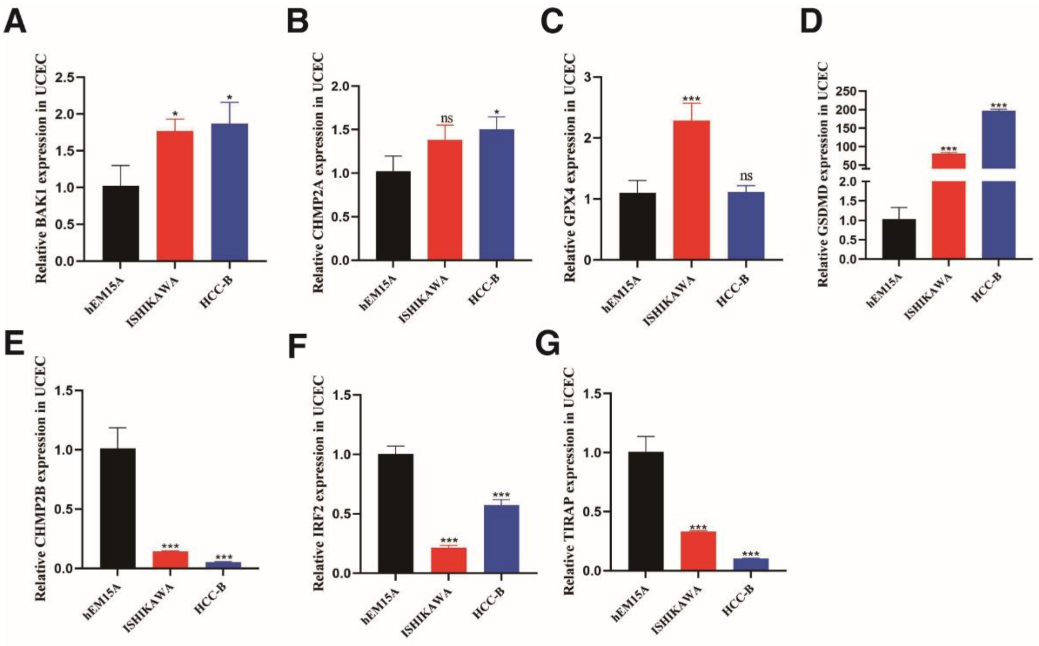

4. **Figure S4:** (A) Boxplots present significant differences in the expression of individual genes across the signature in high- and low-risk groups. (B) Kaplan-Meier curves for the OS of patients in the high- and low-expression of 7 prognosis genes.

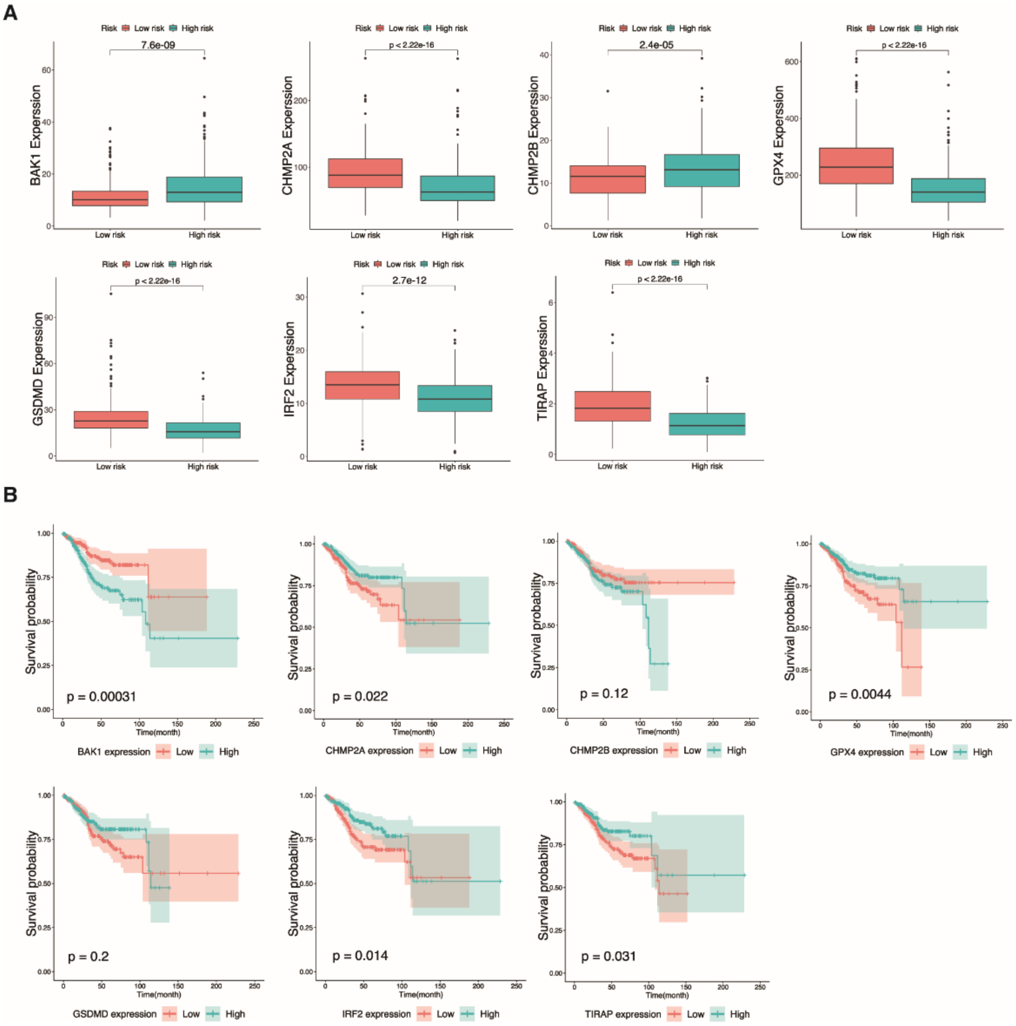

5. **Figure S5:** (A) Relative proportions of each immune cell in TCGA cohort of 532 samples. (B) The correlation plot of immune cells in the UCEC samples.

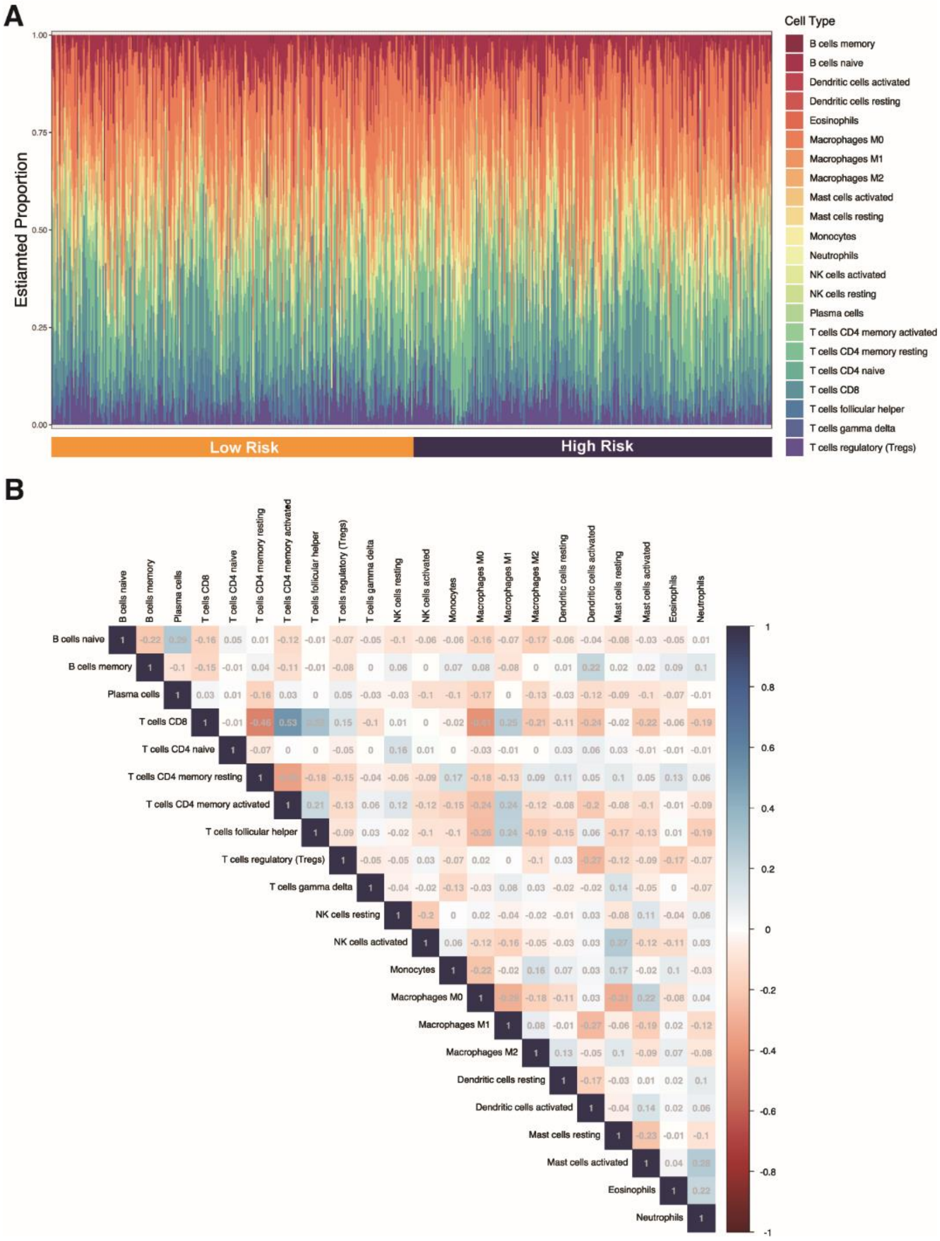

TABLES

All supplementary tables

TableS1: List of genes related to pyroptosis.

Gene	Full-names
AIM2	Absent In Melanoma 2
APIP	Apaf1 Interacting Protein
BAK1	Bcl2 Antagonist/Killer 1
BAX	Bcl2 Associated X, Apoptosis Regulator
CASP1	Cysteine-Aspartic Acid Protease-1
CASP3	Cysteine-Aspartic Acid Protease-3
CASP4	Cysteine-Aspartic Acid Protease-4
CASP5	Cysteine-Aspartic Acid Protease-5
CASP6	Cysteine-Aspartic Acid Protease-6
CASP8	Cysteine-Aspartic Acid Protease-8
CASP9	Cysteine-Aspartic Acid Protease-9
CHMP2A	Charged Multivesicular Body Protein 2A
CHMP2B	Charged Multivesicular Body Protein 2B
CHMP3	Charged Multivesicular Body Protein 3
CHMP4A	Charged Multivesicular Body Protein 4A
CHMP4B	Charged Multivesicular Body Protein 4B
CHMP4C	Charged Multivesicular Body Protein 4C
CHMP6	Charged Multivesicular Body Protein 6
CHMP7	Charged Multivesicular Body Protein 7
CYCS	Cytochrome C, Somatic
DHX9	Dexh-Box Helicase 9
ELANE	Elastase, Neutrophil Expressed
GPX4	Glutathione Peroxidase 4
GSDMA	Gasdermin A
GSDMB	Gasdermin B
GSDMC	Gasdermin C
GSDMD	Gasdermin D
GZMA	Granzyme A
HMGB1	High Mobility Group Box 1
IL18	Interleukin 18
IL1A	Interleukin 1 Alpha
IL1B	Interleukin 1 Beta
IL6	Interleukin 6
IRF1	Interferon Regulatory Factor 1
IRF2	Interferon Regulatory Factor 2
NAIP	Nlr Family Apoptosis Inhibitory Protein
NLRC4	Nlr Family Card Domain Containing 4
NLRP1	Nlr Family Pyrin Domain Containing 1
NLRP2	Nlr Family Pyrin Domain Containing 2
NLRP3	Nlr Family Pyrin Domain Containing 3
NLRP6	Nlr Family Pyrin Domain Containing 6
NLRP7	Nlr Family Pyrin Domain Containing 7
NLRP9	Nlr Family Pyrin Domain Containing 9
NOD1	Nucleotide Binding Oligomerization Domain Containing 1
NOD2	Nucleotide Binding Oligomerization Domain Containing 2
PJVK	Pejvakin/Deafness, Autosomal Recessive 59

PLCG1	Phospholipase C Gamma 1
PRKACA	Protein Kinase Camp-Activated Catalytic Subunit Alpha
PYCARD	Pyd And Card Domain Containing
SCAF11	Sr-Related Ctd Associated Factor 11
TIRAP	Tir Domain Containing Adaptor Protein
TNF	Tumor Necrosis Factor
TP53	Tumor Protein P53
TP63	Tumor Protein P63
ZBP1	Z-Dna Binding Protein 1
CARD8	Caspase Recruitment Domain Family Member 8
DPP8	Dipeptidyl Peptidase 8
DPP9	Dipeptidyl Peptidase 9
SIRT1	Sirtuin 1
STAT3	Signal Transducer And Activator Of Transcription 3
TREM2	Triggering Receptor Expressed On Myeloid Cells 2

TableS2: Parameter settings for the classifier model.

Classifiers	Parameters (Parameters not listed are default)
KNN	n_neighbors=11
logistic regression	metric='l2'
SVM	default
ANN	mait=1000, size=3
decision tree	default
random forest	n_estimators=1000
XGBoost	nrouds=50, eta=0.4, max_depth=3
LightGBM	nrouds=100, metric='l2'
CatBoost	iteration=1000, loss_function='Logloss', learing_rate=0.01

Table S3: Primer sequences for real-time fluorescent quantitative PCR.

Gene Name	Primer sequnces
Actin	Forward: CATGTACGTTGCTATCCAGGC
	Reverse: CTCCTTAATGTCACGCACGAT
BAK1	Forward: GCAGGCTGATCCCGTCC
	Reverse: CTGCGGAAAACCTCCTCTGT
CHMP2A	Forward: AAGCAAGGCCAGATGGATGCT
	Reverse: GGCCTTGGTGACACCCTTCA
CHMP2B	Forward: GCCAAACAACTTGTGCATCTACGG
	Reverse: ACTGCCTGCATTGTTTTTGCTGT
GPX4	Forward: ATGGTTAACCTGGACAAGTACC
	Reverse: GACGAGCTGAGTGTAGTTTACT
GSDMD	Forward: GAGTGTGGCCTAGAGCTGG
	Reverse: GGCTCAGTCCTGATAGCAGTG
IRF2	Forward: AGTGTGCCCAGCGATGAAGA
	Reverse: GAGCTGTTGTAAGGCACCGG
TIRAP	Forward: GCCTTTCACAGGAGAAGTGTCA
	Reverse: ATGTAAGGCCGTAGTGACAGAG

Table S4: Predicted and actual results of different classifiers.

Models	Groups	n	Predicted tumor	True tumor	Predicted normal	True normal
KNN	Testing	84	49	50	34	34
	Validation 1	12	5	7	4	5
	Validation 2	91	58	79	12	12
L2 Logistic	Testing	84	50	50	33	34
Regression	Validation 1	12	7	7	4	5
	Validation 2	91	70	79	12	12
SVM	Testing	84	50	50	33	34
	Validation 1	12	7	7	3	5
	Validation 2	91	74	79	10	12
ANN	Testing	84	50	50	34	34
	Validation 1	12	5	7	4	5
	Validation 2	91	63	79	12	12
Decision Tree	Testing	84	50	50	33	34
	Validation 1	12	7	7	2	5
	Validation 2	91	67	79	2	12
Random	Testing	84	50	50	33	34
Forest	Validation 1	12	7	7	4	5
	Validation 2	91	60	79	3	12
XGBoost	Testing	84	50	50	33	34
	Validation 1	12	6	7	3	5
	Validation 2	91	66	79	3	12
LightGBM	Testing	84	49	50	32	34
	Validation 1	12	7	7	3	5
	Validation 2	91	61	79	2	12
CatBoost	Testing	84	50	50	34	34
	Validation 1	12	7	7	3	5
	Validation 2	91	68	79	5	12

Table S5: TCGA-UCEC clinical parameters.

Characteristic	TCGA-UCEC cohort (n = 532)	
	number	%
**Age**		
<=65	299	56.2
>65	233	43.8
**Histological grade**		
G1	98	18.4
G2	118	22.2
G3	305	57.3
High grade	11	2.1
**Clinical stage**		
Stage I	331	62.2
Stage II	50	9.4
Stage III	122	22.9
Stage IV	29	5.5
**Disease type (primary diagnosis)**		
Adenomas and Adenocarcinomas	395	74.2

Cystic, Mucinous and Serous Neoplasms	137	25.8
**Outcome**		
Alive	444	83.5
Dead	88	16.5

Table S6: Top 20 compounds most related to the PRGRS.

Gene	Drug	Cor	pvalue
IRF2	TP-3654	0.49612126	6.44E-05
GSDMD	Fludarabine	0.43240309	0.00062595
CHMP2B	Vandetanib	-0.4133905	0.00113687
CHMP2B	VE-821	-0.3979439	0.00180072
IRF2	S-49076	0.39689897	0.00185617
IRF2	ZM-336372	0.39164558	0.00215878
CHMP2B	AZD-6738	-0.3898623	0.00227109
CHMP2B	Erlotinib	-0.3842028	0.00266281
GSDMD	AZD-3965	-0.3840579	0.00267358
GPX4	XL-147	0.3829176	0.00275973
IRF2	Nelarabine	0.37925881	0.00305303
GSDMD	Cladribine	0.37830328	0.00313405
GSDMD	Danusertib	-0.3779715	0.00316262
IRF2	SCH-900776	0.37775218	0.00318163
TIRAP	Tivantinib	0.37738105	0.00321404
CHMP2A	GNE-140	-0.3767054	0.0032738
IRF2	Dasatinib	-0.3753545	0.00339622
CHMP2B	Rabusertib	-0.3734727	0.00357352
BAK1	CCT-245737	0.37338631	0.00358186
IRF2	Altiratinib	0.37299858	0.00361947
IRF2	Lestaurtinib	0.3724378	0.00367448
GSDMD	5-Fluoro deoxy uridine 10mer	0.37229383	0.00368872
GPX4	Sabutoclax	0.37198668	0.00371927
IRF2	MK-8033	0.36891229	0.00403761
GPX4	Selumetinib	-0.3655146	0.00441721
IRF2	Bortezomib	0.36405578	0.00458963
BAK1	5-Fluoro deoxy uridine 10mer	0.35937574	0.00518346
IRF2	Fluphenazine	0.35899153	0.00523508
GPX4	Pimasertib	-0.3558409	0.00567576
CHMP2B	RG-7741	-0.3549152	0.00581129
